# Compression Eliminates Charge Traps by Stabilizing Perovskite Grain Boundary Structures: An Ab Initio Analysis with Machine Learning Force Field

**DOI:** 10.1021/acs.chemmater.3c03261

**Published:** 2024-03-12

**Authors:** Dongyu Liu, Yifan Wu, Mikhail R. Samatov, Andrey S. Vasenko, Evgueni V. Chulkov, Oleg V. Prezhdo

**Affiliations:** † HSE University, 101000 Moscow, Russia; ‡ Department of Chemistry, University of Southern California, Los Angeles California 90089, United States; § Donostia International Physics Center (DIPC), 20018 San Sebastián - Donostia, Euskadi, Spain; ∥ Centro de Física de Materiales (CFM-MPC), Centro Mixto CSIC-UPV/EHU, 20018 San Sebastián - Donostia, Euskadi, Spain; ⊥ Departamento de Polímeros y Materiales Avanzados: Física, Química y Tecnología, Facultad de Ciencias Químicas, Universidad del País Vasco UPV/EHU, 20080 San Sebastián - Donostia, Euskadi, Spain; # Department of Physics & Astronomy, University of Southern California, Los Angeles California 90089, United States

## Abstract

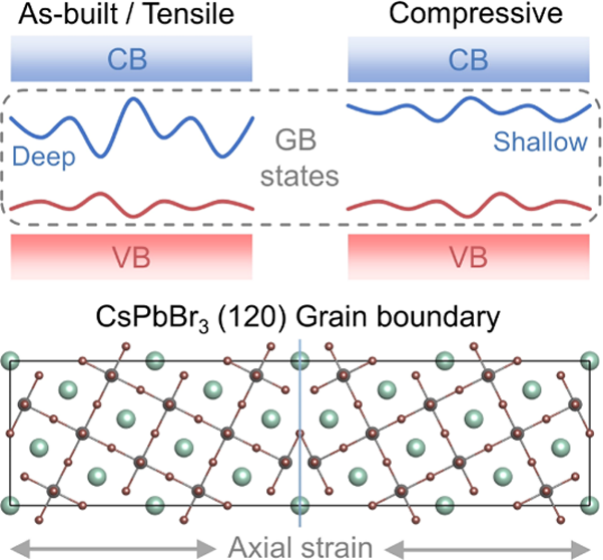

Grain boundaries (GBs) play an important role in determining the optoelectronic properties of perovskites, requiring an atomistic understanding of the underlying mechanisms. Strain engineering has recently been employed in perovskite solar cells, providing a novel perspective on the role of perovskite GBs. Here, we theoretically investigate the impact of axial strain on the geometric and electronic properties of a common CsPbBr_3_ GB. We develop a machine learning force field and perform ab initio calculations to analyze the behavior of GB models with different axial strains on a nanosecond time scale. Our results demonstrate that compressing the GB efficiently suppresses structural fluctuations and eliminates trap states originating from large-scale distortions. The GB becomes more amorphous under compressive strain, which makes the relationship between the electronic structure and axial strain nonmonotonic. These results can help clarify the conflicts in perovskite GB experiments.

## Introduction

Metal halide perovskites (MHPs) are promising candidates for the next generation solar cells because of their excellent optoelectronic properties and cost-efficient solution processability.^1−5^ The record power conversion efficiency (PCE) of MHP-based solar cells has grown rapidly from 3.8% in 2009 to 26.1% today, approaching the record PCE of silicon-based solar cells.^6,7^ To further improve the performance of MHPs, recent work focuses on passivating the harmful structural defects that serve as carrier recombination centers.^8−18^ Despite the “defect tolerance” property of MHPs,^19−22^ the carrier recombination at grain boundaries (GBs) is still observed to be relatively fast, and the PCE is therefore limited.^23−25^ Besides, GBs are also reported to assist ion migration in MHPs and result in stability issues.^26^ Numerous strategies have been developed to mitigate the detrimental influence of GBs in MHP-based solar cells, such as saturating dangling bonds at the boundaries and enlarging grain size to reduce the GB density.^27−34^ However, the mechanism by which GBs affect the MHP performance remains unclear,^35,36^ and GBs even show positive effects under some conditions.^37−39^ These problems are mainly contributed by the complexity and diversity of MHP GBs, and the lack of a fundamental understanding of the GB behavior severely hinders the investigation of novel passivation techniques.

In addition to experimental characterization, ab initio calculations are widely employed to study the electronic properties of MHP GBs using atomistic models.^40^ Neither pristine nor defective MHP GBs are found to introduce deep trap states in the forbidden band,^41−43^ which is consistent with the aforementioned “defect tolerance”. However, some defective configurations in the GB region produce localized electronic states around the band edge, accelerating the interband carrier recombination by enhancing the electron-vibrational nonadiabatic couplings (NACs) compared with bulk MHPs.^44−50^ Moreover, GBs are identified to accumulate point defects because of their relatively low formation energies, and the subsequent structural distortions can make these defect states deeper.^51−55^ These results indicate a complex correlation between the geometric structure of MHP GBs and their impact on the optoelectronic performance. Furthermore, given the soft lattice of MHPs,^56−58^ thermal fluctuations are reported to produce large-scale distortions at vacancy sites.^59^ Our recent work demonstrates that GBs also suffer from such large-scale structural fluctuations, and the distorted structures create localized trap states owing to the Pb–Pb interactions across the boundaries.^60,61^ Trap states are dynamically generated under thermal fluctuations, even in stoichiometric GBs, and this can be an important source of carrier recombination centers at GBs. Previous studies have suggested that suppressing the structural distortions of GBs represents a promising direction to eliminate these recombination centers.

In this work, we theoretically investigate the impact of axial strain on the geometric and electronic properties of the ∑5 (120) GB in a CsPbBr_3_ MHP. We construct tensile-strain, strain-free, and compressive-strain GB models by adjusting the axial length according to the calculated energy–strain diagram. Such strain can be achieved experimentally by choosing suitable substrate materials and operating temperatures, and a positive correlation between the solar cell performance and the compressive strain has been reported.^62^ In particular, because MHPs usually exhibit higher thermal expansion coefficients than inorganic carrier transport layer materials, tensile strain is likely to be introduced when samples are prepared at relatively high temperatures and cooled to room temperature. Indeed, strain in perovskites has been widely studied because it dramatically affects performance, including efficiency and stability.^63,64^ However, most studies focus on the lattice properties, while an understanding of the interplay between strain and perovskite GBs is still lacking. Considering that the GBs may lead to more defects and serve as ion-migration channels, it is necessary to investigate the influence of strain on perovskite GBs. We perform molecular dynamics (MD) simulations to sample atom movements at ambient temperature and calculate the electronic structure by using ab initio density functional theory (DFT). With the help of the developed machine learning (ML) force field (FF), we analyze the evolution of the geometric and electronic structures of GB models on a nanosecond time scale, which is comparable to the carrier recombination time in CsPbBr_3_ and is much longer than that in ab initio MD (AIMD) simulations.^46,65^ The tensile-strain model exhibits large-scale structural fluctuations and generates localized trap states near the conduction band. We demonstrate that compressing the GB models in the axial direction eliminates the trap states by suppressing the structural distortions. Specifically, the axial strain regulates the local environment of the unsaturated Pb atoms in the GB region, and the enhanced Pb–Br interactions are resistant to structural changes. Further, the compressive strain leads to the reconstruction of the GB toward an amorphous-like configuration, making the relationship between the geometric structure and axial strain nonmonotonic. These results provide new insights into understanding the role of GBs in MHP solar cells.

## Methods

Ab initio DFT calculations were carried out using the Vienna Ab initio Simulation Package (VASP).^66−68^ The Perdew–Burke–Ernzerhof (PBE) functional^69^ together with the projected-augmented wave (PAW) method^70,71^ was used to describe the electron–ion interactions. A cutoff energy of 300 eV was chosen for the plane-wave basis set. The energy convergence criterion for electronic structure calculations was 10^–5^ eV, and the force criterion for structure relaxation was 0.02 eV/Å. The dispersion interactions were considered with Grimme’s D3 model.^72,73^ We built ∑5 (120) GB models by combining two CsPbBr_3_ (120) slabs (cubic phase) in opposite directions using the Atomsk code,^74^ and we changed the axial length to introduce strain. The four-layer slabs were adopted to prevent the interactions between two GBs in the periodic cell.^42^ Moreover, we doubled the slab models along the [001] direction to consider the sliding effect.^61^ The GB models comprise multiple unit cells and contain 200 atoms. Only the Γ-point of the Brillouin zone was used, which reduces the computational cost and minimizes the unphysical interactions between the periodic images of the GBs arising due to the finite system size. The ML FF was trained with the DeePMD-kit package^75^ and implemented in the LAMMPS code for MD simulations.^76^ We used the se_e2_a descriptor with a cutoff radius of 9 Å to construct the neural network potential. The dimensions of the embedding and fitting layers were 25 × 50 × 100 and 240 × 240 × 240, respectively. We trained one ML FF for all of the GB models, and its accuracy was verified by comparing the ML-predicted potential energies with the DFT calculation results (RMSE < 10 meV/atom) as shown in Figure S1. All MD simulations were performed at 300 K in a canonical ensemble. The visualization of structures was accomplished with the VESTA software package.^77^

## Results and Discussion

**Figure 1:**
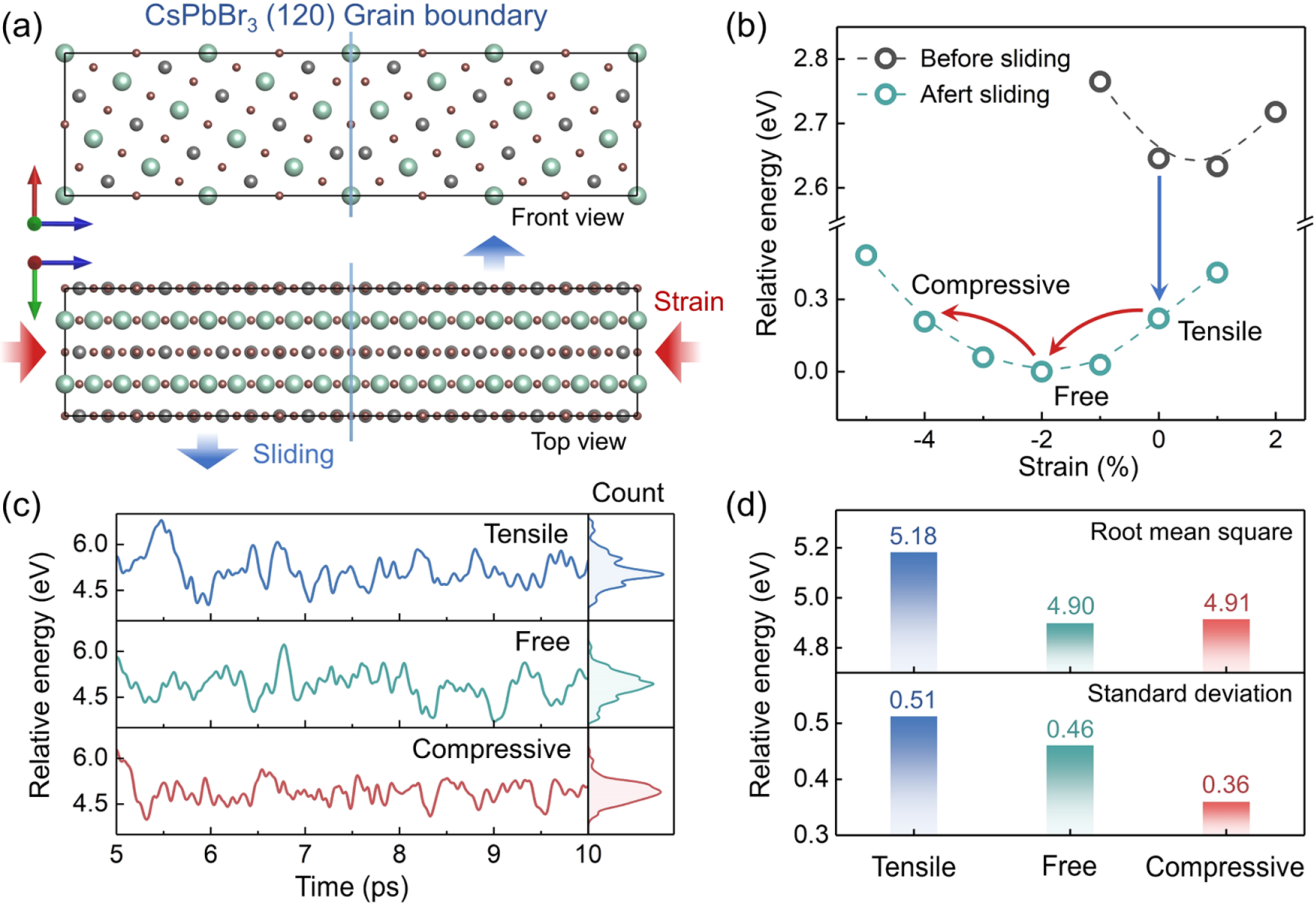
(a) Front and top views of the CsPbBr_3_ GB model. Color codes: green for Cs, gray for Pb, and brown for Br. (b) Relative energy change of the GB system with respect to sliding and strain. (c) Relative potential energy fluctuation of different GB models in the last 5 ps of 10 ps AIMD trajectories. (d) Root mean square and standard deviation of the relative potential energy results in the last 5 ps trajectories.

Figure 1a shows the structure of the as-built ∑5 (120) CsPbBr_3_ GB model. This structure corresponds to a mirror-symmetric twin GB composed of two CsPbBr_3_ grains along the (120) crystal surface. ∑5 represents for the number of lattice points in a unit cell of the coincidence site lattice of the GB, determining a tilt angle of 36.9°. Our previous work demonstrates that this system is in a metastable state, and the GB spontaneously slides in the direction of the front view within several picoseconds in MD simulations.^61^ The sliding changes the interaction between the lateral grains, breaks the original equilibrium structure, and induces stress in the axial direction. Figure 1b displays the relative energy changes of the GB model with respect to the sliding and strain effects, where the atom positions are fully relaxed but the size of the simulation cell is fixed. We introduce the strain by changing the axial length with steps of 1%. The initial structure lies close to the minimum of the energy curve before sliding because the lattice constant is from the optimized bulk CsPbBr_3_. Sliding dramatically reduces the system energy, and the positive slope of the energy curve after sliding indicates the presence of a tensile strain in the model. After reducing the axial length by 2%, the sliding system is close to the minimum of the energy curve and becomes strain-free. Moreover, we further impose a −2% length change to consider the compressive-strain condition. It should be pointed out that such strains are comparable to those observed in experiments.^62^ AIMD simulations are carried out to investigate the impact of strain on structural fluctuations. We ran 10 ps AIMD trajectories for each GB model. The first 5 ps is for heating the system to the equilibrium state, and the last 5 ps trajectories are used for analysis. Figure 1c,d exhibits the potential energy fluctuations and the statistical results, respectively. The root mean square (RMS) of the potential energy decreased in the tensile-strain model compared to the strain-free model, which is consistent with the energy–strain diagram. However, the compressive strain only slightly increases the potential energy, and the fluctuation is suppressed, as indicated by the standard deviation changes. These results demonstrate that the strain in the CsPbBr_3_ GB not only affects the interatomic potential energy but also interferes with the structural oscillation.

**Figure 2:**
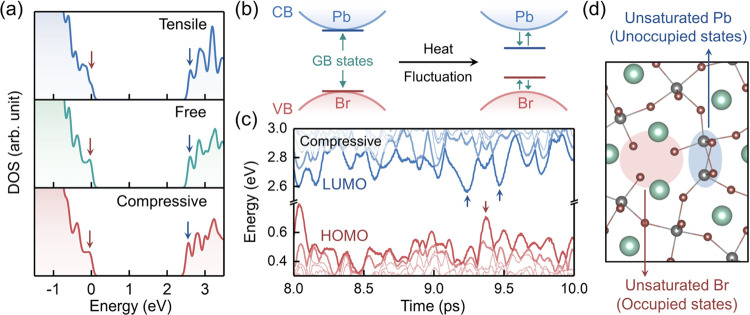
(a) Density of states (DOS) plots of the GB models. Red and blue arrows indicate the band edge states. (b) Schematic of the changes in the GB electronic structure under thermal fluctuations. (c) Energy level evolution of the compressive-strain model in the last 2 ps of the 10 ps AIMD trajectory. (d) Structures of the unsaturated Pb and Br atoms in the GB region.

The density of states (DOS) of different GB models is plotted in Figure 2a. The GB structure creates electronic states near the band edge, which are rarely affected by the strain (Figure S2). Nevertheless, the CsPbBr_3_ GB exhibits large-scale motions under thermal fluctuations, and the structural distortion makes these GB states deep in the forbidden band.^61^Figure 2b illustrates a scheme of this process, and the energy level evolution of the compressive-strain GB model in the last 2 ps is illustrated in Figure 2c as an example (the full results are given in Figure S3). The highest occupied molecular orbital (HOMO) and lowest unoccupied molecular orbital (LUMO) can be isolated from other orbitals during oscillations, as indicated by the arrows. Such electronic states are expected to trap photogenerated carriers and facilitate their recombination, leading to energy losses. Figure 2d shows the optimized structure of the CsPbBr_3_ GB after sliding. Unsaturated Pb and Br atoms are formed at the boundaries and are responsible for the GB states at the band edge. When the GB structure is distorted, the distance between Br atoms can be shortened and electrostatic repulsion makes the system less stable, raising the energy level of HOMO. On the other hand, when Pb atoms become closer, the hybridization between their empty 6p orbitals is enhanced, lowering the energy level of the LUMO. These two effects together contribute to the appearance of midgap trap states. Therefore, suppressing the structural distortion represents a promising approach to prevent the formation of these trap states.

**Figure 3:**
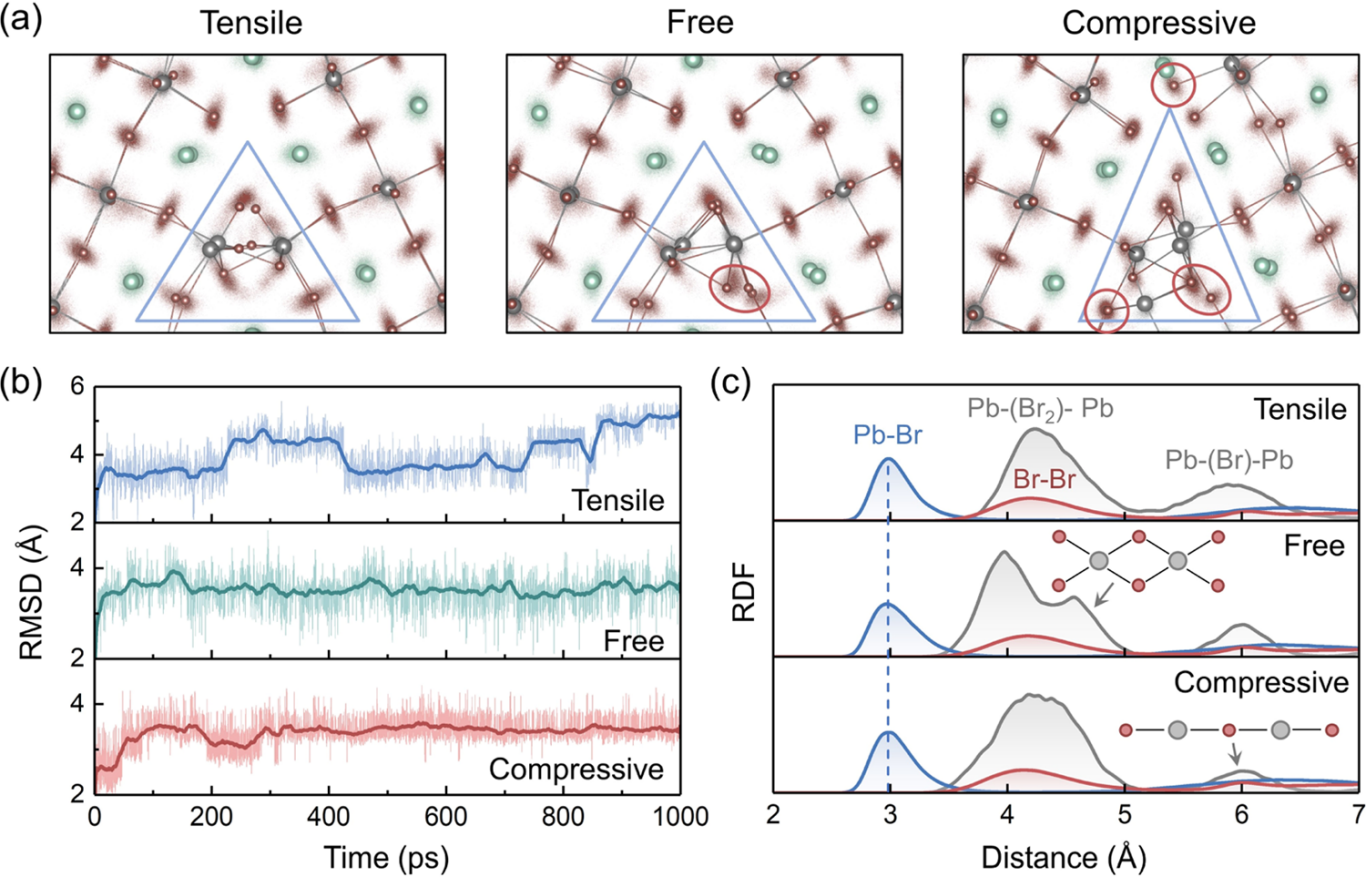
(a) Structural oscillations of the GB models in the 1 ns MLMD trajectories. The clouds represent the atomic distributions in the MLMD trajectories, and the time-averaged structures are shown by the ball-and-stick models. GB distortion and Br atom migration are indicated by the blue triangles and red circles, respectively. (b) Root-mean-square displacement (RMSD) evolution of the GB models in the MLMD trajectories. The thick lines display the moving averages over 20 ps. (c) Time-averaged radial distribution functions (RDFs) of the Pb and Br atoms in the GB region in the MLMD trajectories.

The carrier lifetime in CsPbBr_3_ is reported at the nanosecond time scale,^65^ and some slow fluctuations and rare events may occur in this period,^59−61^ creating trap states and promoting carrier recombination. However, AIMD simulations are limited to several picoseconds because of costly quantum mechanics calculations. To address this time scale issue, ML FF is developed to predict the interatomic interactions directly from the local configurations. We perform 1 ns ML-based MD (MLMD) simulations for each GB model to obtain the geometric structure evolution, and we calculate the energy levels every 1 ps with DFT to track the electronic structure evolution. Figure 3a displays the distribution of the atoms in the 1 ns MLMD trajectories (scatter plot) and their average positions (ball-and-stick model) in the GB region. The results of all of the models are given in Figure S4. The atoms in the bulk region mainly oscillate around the equilibrium positions, while the GB configuration, especially in the blue triangles, is modified by the axial strain. Specifically, the adjacent Br atoms migrate to coordinate with the unsaturated Pb atoms, as indicated by the brown circles, and the original Pb–Pb coordination is broken in the compressive-strain model. Figure 3b shows the evolution of the root-mean-square displacement (RMSD) for all of the atoms in the different GB models. The tensile-strain model exhibits slow structural fluctuations with a period of several hundred picoseconds, while such fluctuations are effectively suppressed in the strain-free and compressive-strain models. Besides, the RMSD curves also indicate that structural distortions in the strain-free and compressive-strain models occur within 100 ps, which is much faster than carrier recombination. The structural changes are further investigated with a radial distribution function (RDF) as shown in Figure 3c. We calculate the RDF between the Pb atoms in the GB region (i.e., the blue triangular region) and the Br atoms coordinated to them in the initial structure. The axial strain mainly affects the Pb–Pb coordination, while the localized Pb–Br interaction is nearly maintained. The Pb–Pb RDF can be divided into two parts (around 4 and 6 Å), which correspond to the Pb atoms separated by two and one Br atoms, respectively. Compressing the tensile-strain model in the axial direction increases the ratio of the first part of the Pb–Pb RDF curve, indicating a structural transition from the initial perovskite structure to the distorted GB configuration. This deduction is consistent with the structural changes observed in Figure 3a. Moreover, the compressive strain leads to a more uniform distribution of the Pb–Pb RDF around 4 Å, implying the appearance of an amorphous-like structure. Compared with the RMSD evolution results, such strain-induced reconstruction of the GB enhances its resistance toward structural fluctuations.

**Figure 4:**
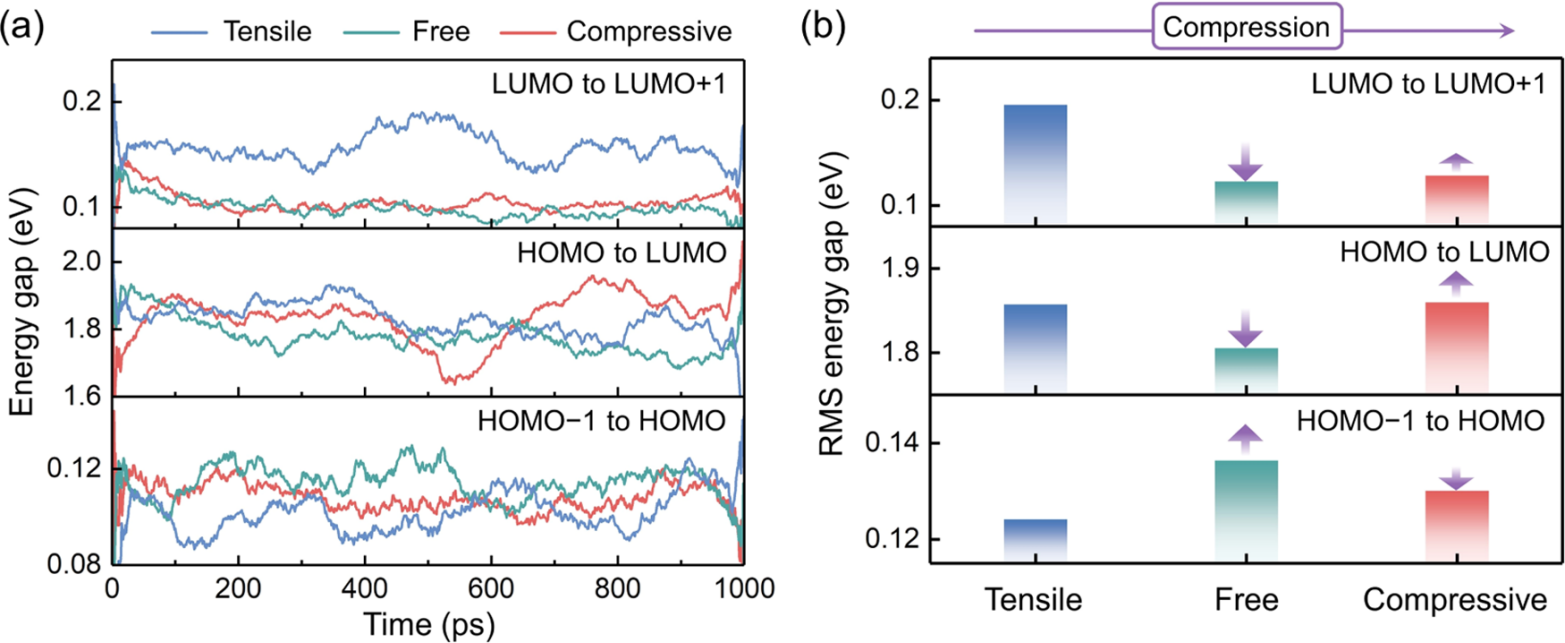
(a) Energy gap evolution of the GB models in the MLMD trajectories. The plotted data are calculated as the moving averages over 100 ps. (b) Root mean square (RMS) of the energy gaps in the MLMD trajectories.

Figure 4a shows the energy gap evolution in the MLMD trajectories. The moving average results are plotted to illustrate their trends, and the raw results can be found in Figures S5–S7. AIMD simulations demonstrate that the HOMO and LUMO can become deep in the forbidden band under thermal fluctuations. In the long MLMD trajectories, only the tensile-strain model exhibits a relatively large energy gap from LUMO to LUMO + 1. Such discrepancy can be attributed to the time-scale limitations of AIMD. On the one hand, the reconstruction of the GB is relatively slow, and the fluctuation suppression cannot be observed in short trajectories. On the other hand, the deep HOMO appears much less frequently than the deep LUMO, which makes the moving average of the energy gap between HOMO – 1 and HOMO small. The LUMO in the CsPbBr_3_ GB is mainly contributed by the unsaturated Pb atoms in the boundary region, and the Pb–Pb interactions across the boundary can lower the energy level. Nevertheless, compressing the tensile-free model modifies the Pb–Pb coordination at the GB region and suppresses the relevant structural oscillations, thus preventing the formation of deep LUMO levels. The Br–Br and Pb–Br interactions are rarely affected by the axial strain, and the HOMO – 1 to HOMO and HOMO to LUMO gaps are less changed. Figure 4b illustrates the root mean square (RMS) of the energy gaps in different GB models. Releasing the tensile strain not only eliminates the deep LUMO states in the strain-free model but also decreases the HOMO–LUMO gap and moves the HOMO toward the forbidden band. The narrowed bandgap can accelerate interband carrier recombination, and the isolated HOMO may trap holes, which are detrimental to solar cells. Moreover, if the axial length is further reduced to induce compressive strain, the HOMO–LUMO gap and the HOMO level are partially recovered compared to the strain-free model, exhibiting a nonmonotonic dependence on the axial strain. Furthermore, the energy gap from LUMO to LUMO + 1 shows only a slight increment in the compressive-strain model since the LUMO level is dominated by the strain-induced GB reconstruction.

**Figure 5:**
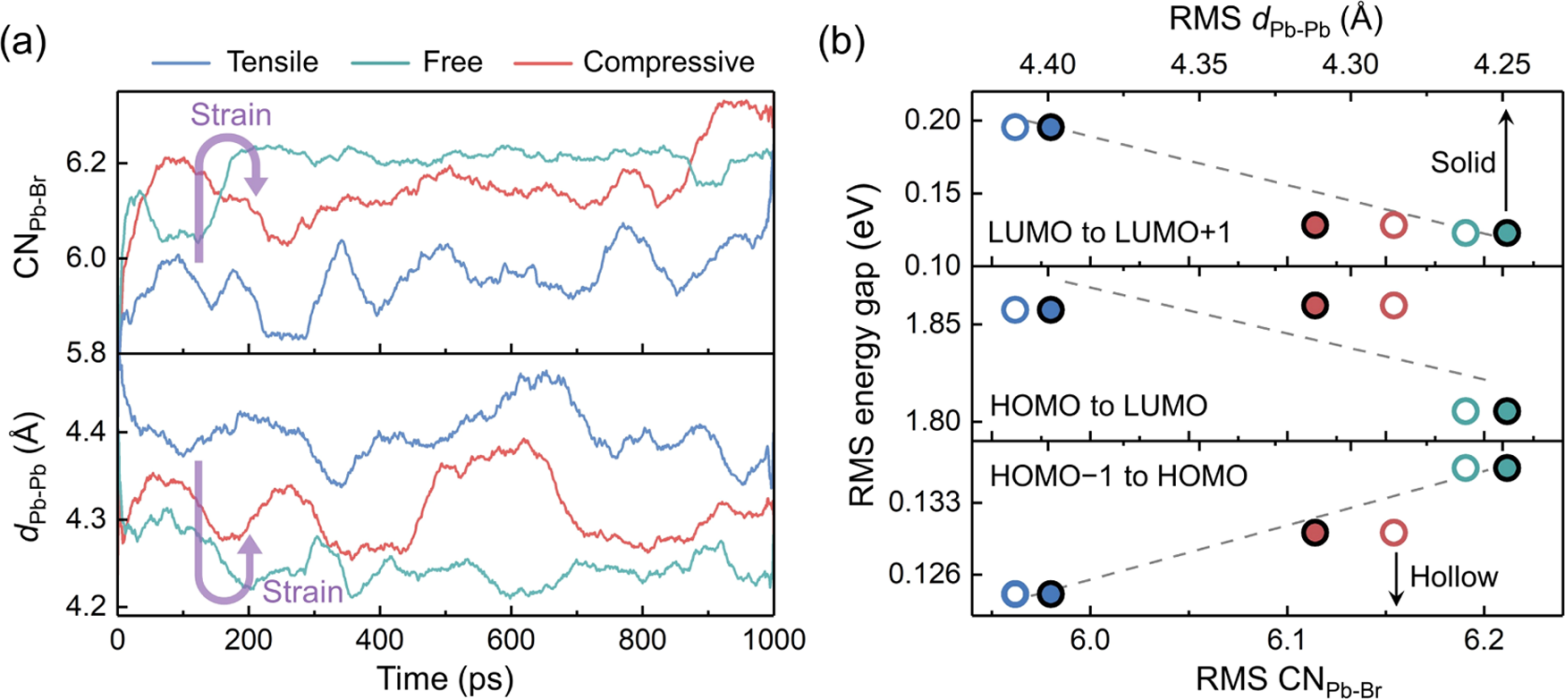
(a) Evolutions of the Pb–Br coordination number (CN_Pb–Br_) and the Pb–Pb distance (*d*_Pb–Pb_) in the GB region in the different models along the MLMD trajectories. The plotted data are calculated as the moving average over 50 ps. (b) Dependence of the root mean square (RMS) of the energy gaps on the RMS of CN_Pb–Br_ and *d*_Pb–Pb_ in the different GB models.

In general, strain regulates the electronic structure of perovskites by changing the lattice constant.^78^ Our calculations demonstrate that the axial strain also controls the reconstruction of the CsPbBr_3_ (120) GB under thermal fluctuations, having a more complex influence on the electronic structure. The GB reconstruction mainly involves Br migration toward Pb and the Pb–Pb coordination changes. To quantitatively investigate the impact of these two factors, we calculate the evolutions of the average Pb–Br coordination number (CN_Pb–Br_) and the average Pb–Pb distance (*d*_Pb–Pb_) as plotted in Figure 5a. CN_Pb–Br_ is calculated from the Pb–Br RDF curve with a cutoff of 4 Å, which corresponds to the end of the first coordination shell of Pb. This cutoff is larger than the equilibrium Pb–Br bond distance of 2.97 Å, allowing more than six Br atoms to enter this region in the distorted structures and be counted. Hence, during the MD simulations, CN_Pb–Br_ can be larger than six, which is the ideal value for pristine CsPbBr_3_. The raw results can be found in Figures S8 and S9. Compared with the tensile-strain model, the strain-free model exhibits more Pb–Br interactions and shorter Pb–Pb distances, which are consistent with the compressed configuration with a higher atomic density. However, for the compressive-strain model, although the axial length is further reduced, CN_Pb–Br_ and *d*_Pb–Pb_ change in the reverse direction as indicated by the purple arrows. The structural transition in the compressive-strain model generates amorphous-like structures, which break the compressed GB configuration in the strain-free model and partially recover the Pb–Pb and Pb–Br coordination, resulting in nonmonotonic correlations with the axial strain. Figure 5b demonstrates the dependence of the energy gaps on these structural descriptors. We find linear relationships between them, and the HOMO–LUMO gap shows the same trend as the bandgap in bulk perovskites.^78^ Meanwhile, the high atomic density (i.e., large CN_Pb–Br_ and small *d*_Pb–Pb_) suppresses the LUMO fluctuation but makes the HOMO slightly deep. The compressive-strain model distributes in the middle of this range, which is expected to balance these factors and achieve a better photovoltaic performance.

GBs play an important role in determining the performance of MHP solar cells, and various additives are developed to mitigate their detrimental effects.^79−81^ However, the mechanism by which GB interact with the carriers and ions in MHPs is still under debate. On the other hand, strain engineering has recently been investigated to tune the properties of MHPs in solar cells,^63,64,82^ and the tensile and compressive strains are reported to reduce and improve the PCEs, respectively.^62^ Here, our calculation results demonstrate that the axial strain significantly modifies the GB configuration in CsPbBr_3_ and suppresses the structural fluctuations, preventing the formation of the detrimental trap states owing to the large-scale distortions. Especially, we notice that the compressive strain leads to a transition toward amorphous-like structures in the GB region. Given that the GBs are also reported as ion-migration channels in MHPs,^83^ the strain-induced structural change is expected to impede the ion motions and alleviate the relevant stability issues.^26^ Indeed, the amorphous GBs have been observed in various additive-passivated MHPs,^30,32,84−86^ and such amorphous configurations are identified to be beneficial for MHP solar cells.^23,87^ Therefore, we anticipate that the compressive strain has impacts similar to those of the additives on passivating the MHP GBs, thus improving the solar cell performance. Moreover, since the residual strain widely exists in the practical MHP samples, this mechanism may provide new insights into understanding the conflicts in MHP GB studies.

## Conclusions

To recapitulate, we demonstrate that introducing compressive axial strain efficiently suppresses structural fluctuations in the CsPbBr_3_ (120) GB and eliminates trap states originating from large-scale distortions. DFT calculations indicate that the as-built GB model is in a metastable state and slides spontaneously along the GB direction to lower the system energy. Sliding induces tensile strain in the axial direction according to the energy–strain diagram. We vary the axial length of the GB model to construct strain-free and compressive-strain models. The optimized GB exhibits electronic defect states at the band edge, but these states can become deep in the forbidden band under thermal fluctuations. Such isolated states are thought to trap the photogenerated carriers and accelerate their recombination. Considering that carrier recombination in CsPbBr_3_ occurs over nanoseconds or longer while AIMD simulations are limited to several picoseconds, we combine MLMD and ab initio DFT to obtain the evolution of the geometric and electronic structures over 1 ns. The tensile-strain model exhibits large-scale and slow motions in the long trajectory, and applying axial strain efficiently suppresses such structural fluctuations in the strain-free and compressive-strain models. Meanwhile, the deep LUMO in the tensile-strain model becomes shallow because the additional strain prevents the formation of the corresponding distorted structures. Further, the compressive strain leads to the reconstruction of amorphous structures at the GB, producing a nonmonotonic relationship between the electronic structure and the axial strain. Since compressive strain is reported to be beneficial for MHPs and amorphous GBs are also observed in various additive-passivated MHPs, we propose a mechanism in which strain passivates MHP GBs by breaking the original sensitive structure and transforming the GB configuration into a distortion-resistant amorphous state. This established strain dependence of the GB properties can help in understanding various experimental observations and provides a new perspective for the development of efficient and stable MHPs.

## Supporting Information Available

Verification of the accuracy of the ML FF, charge density distribution of the band edge states, energy level evolution in the AIMD trajectories, atom distribution of entire GB models in the MLMD trajectories, evolution of energy gaps and structural descriptors (i.e., CN_Pb–Br_ and *d*_Pb–Pb_) in the MLMD trajectories (raw data and moving average), and structural information on GB models before and after optimization (PDF)
