# Supplementary material for: Compression Eliminates Charge Traps by Stabilizing Perovskite Grain Boundary Structures: An Ab Initio Analysis with Machine Learning Force Field
Source: Chem Mater. 2024 Mar 12;36(6):2898–906. doi: 10.1021/acs.chemmater.3c03261 (PMC10976646; doi:10.1021/acs.chemmater.3c03261)
Supplement: Supplementary file 1 — cm3c03261_si_001.pdf [file cm3c03261_si_001.pdf]

Supporting Information for

Compression eliminates charge traps by stabilizing perovskite grain boundary structure: ab initio analysis with machine learning force field

Dongyu Liu[1], Yifan Wu[2], Mikhail R. Samatov[1], Andrey S. Vasenko[1,3], Evgueni V. Chulkov[3,4,5*], Oleg V. Prezhdo[2,6*]

[1]HSE University, 101000 Moscow, Russia

[2]Department of Chemistry, University of Southern California, Los Angeles CA 90089, USA

[3]Donostia International Physics Center (DIPC), 20018 San Sebastián - Donostia, Euskadi, Spain

[4]Centro de Física de Materiales (CFM-MPC), Centro Mixto CSIC-UPV/EHU, 20018 San Sebastián - Donostia, Euskadi, Spain

[5]Departamento de Polímeros y Materiales Avanzados: Física, Química y Tecnología, Facultad de Ciencias Químicas, Universidad del País Vasco UPV/EHU, 20080 San Sebastián - Donostia, Euskadi, Spain

[6]Department of Physics & Astronomy, University of Southern California, Los Angeles CA 90089, USA

**Corresponding author E-mail: evguenivladimirovich.tchoulkov@ehu.eus (E. V. C.), prezhdo@usc.edu (O. V. P.)*

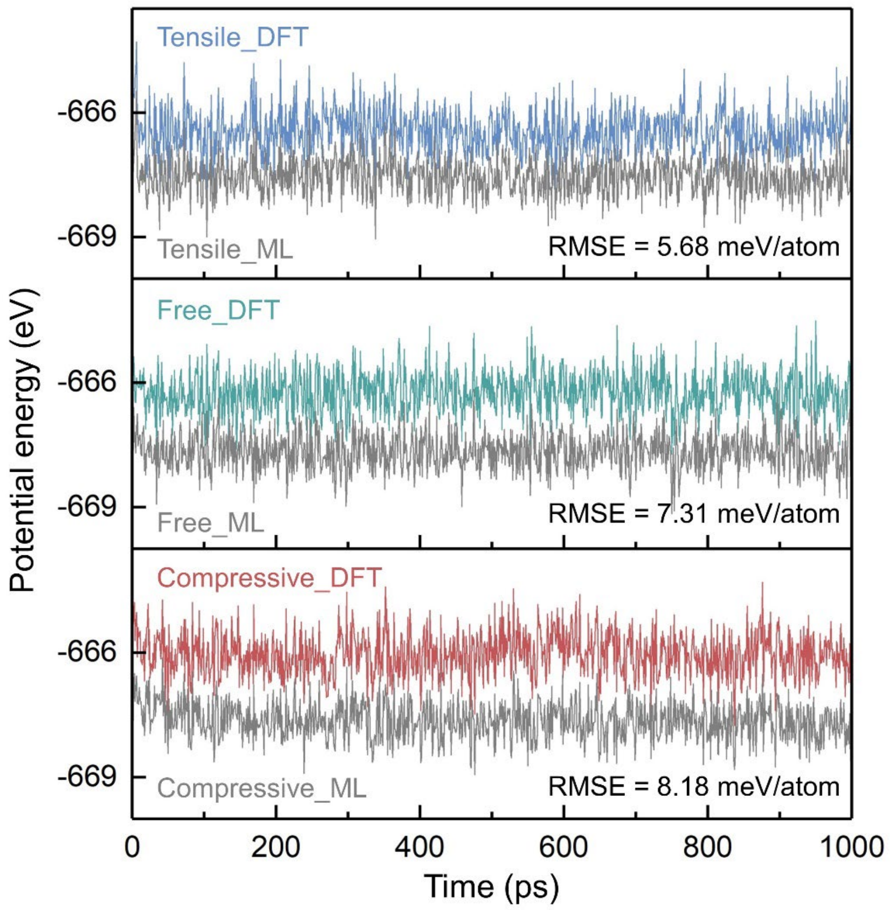

Figure S1. Comparison of the potential energies from DFT calculations and ML predictions in the 1 ns MLMD trajectories. Since the structures in the training sets are mainly from the tensile-strain GB model, the RMSE slightly increases in the strain-free and compressive-strain GB models.

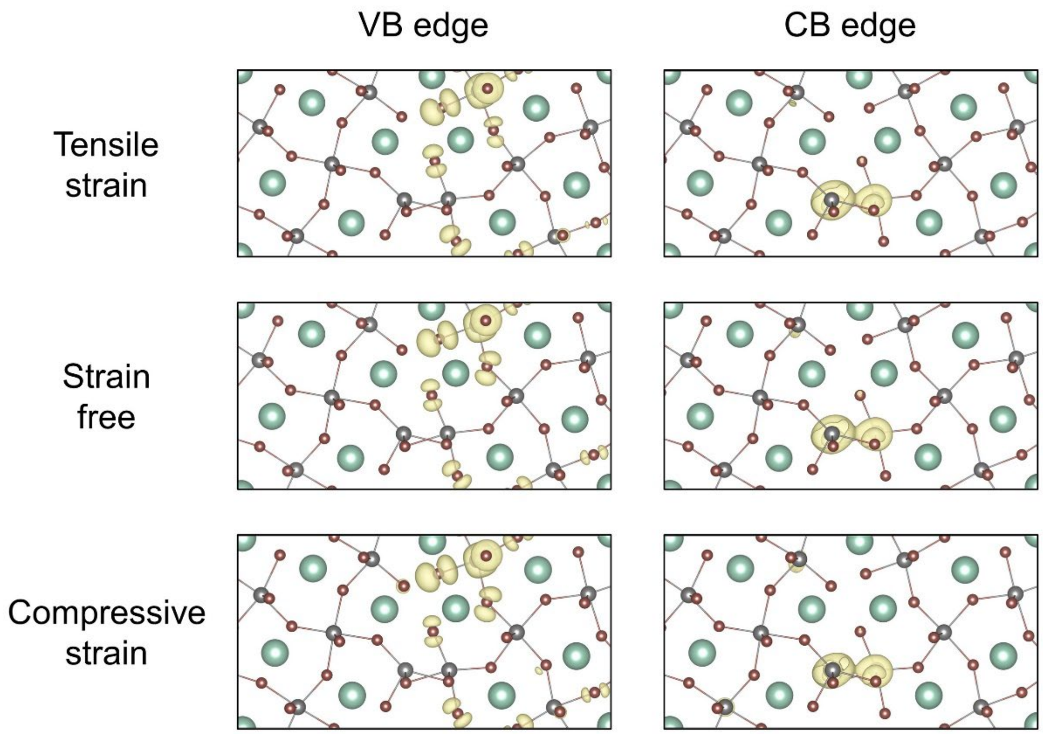

Figure S2. Charge density distributions of the electronic states at the band edges in different GB models. For the CB, there are four nearly degenerate states at the band edge, and the charge densities are averaged over all of them. Only the HOMO is considered at the VB edge.

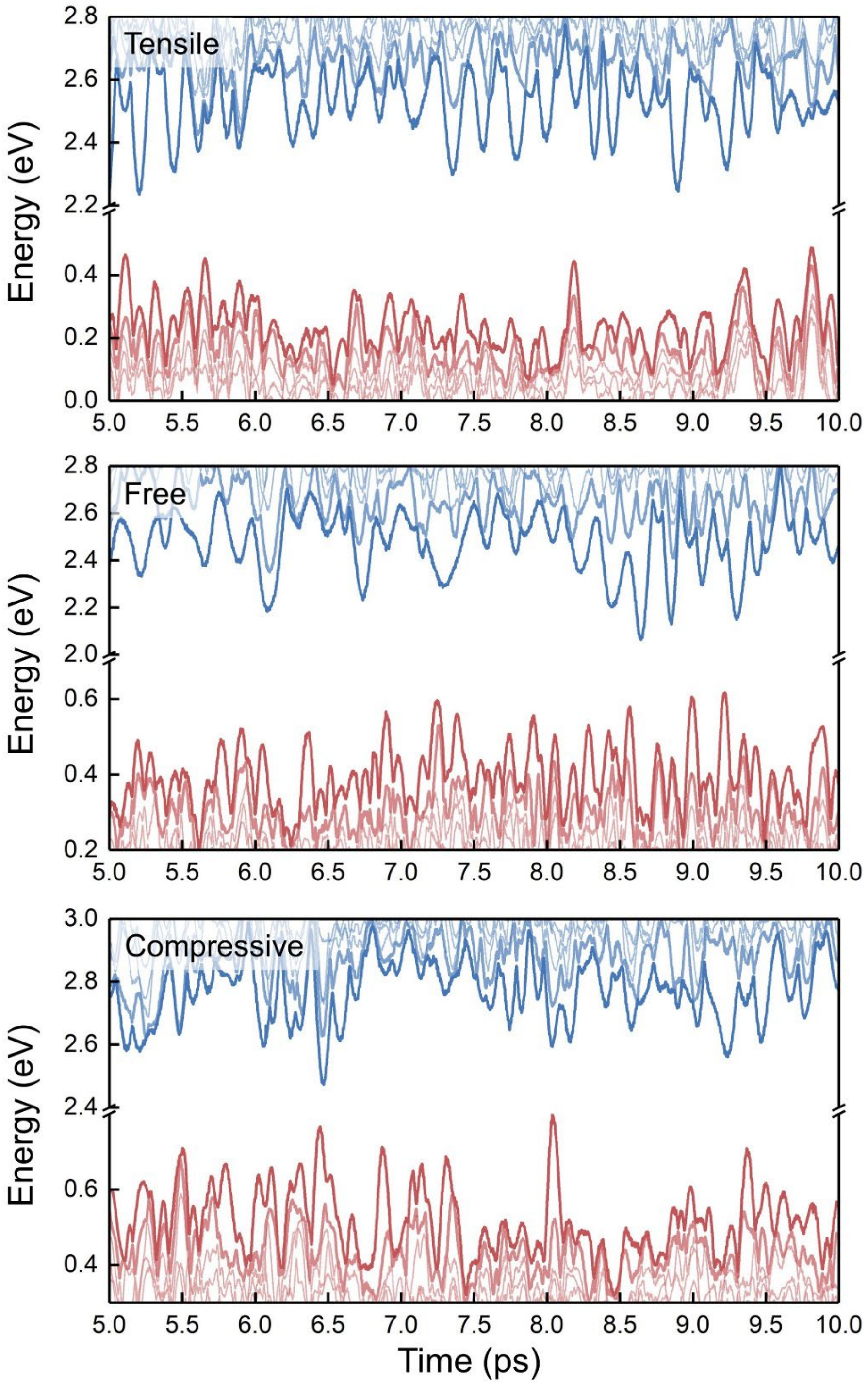

Figure S3. Energy level evolution of different GB models in the last 5 ps of 10 ps AIMD trajectories.

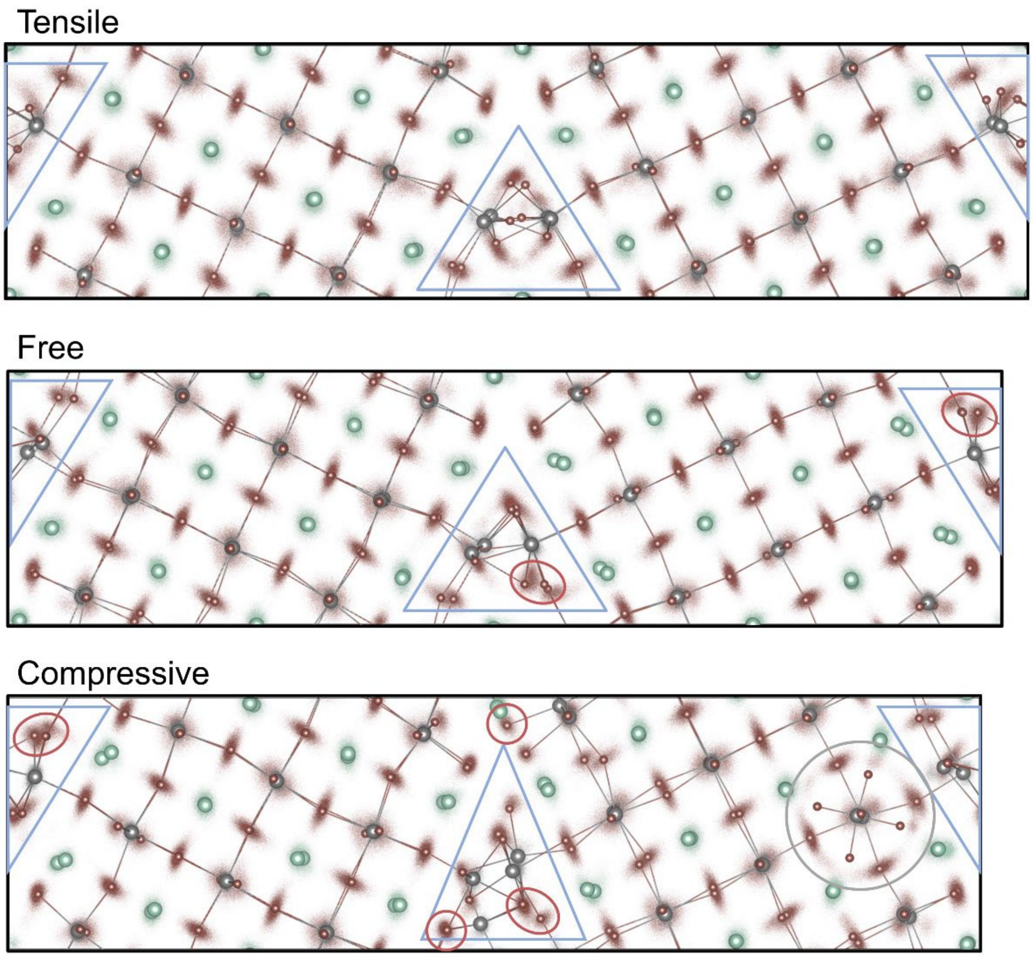

Figure S4. Atom distribution (scatter plot) and average structure (ball-and-stick model) of the GB models in the 1 ns MLMD trajectories. The GB distortion and Br atom migration are indicated by the blue triangles and red circles, respectively. Note that one Pb−Br octahedron rotates once in the compressive-stain model, as shown in the gray circle.

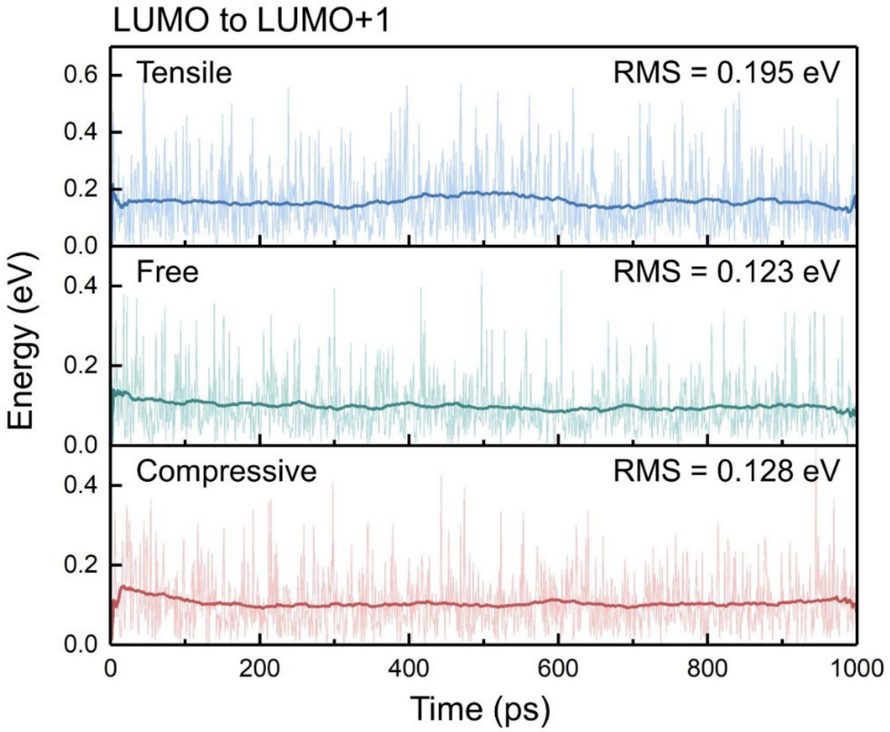

Figure S5. Evolution of the energy gap from LUMO to LUMO+1 in the GB models. The thin lines are from DFT calculations with a timestep of 1 ps, and the thick lines indicate the moving average over 100 ps.

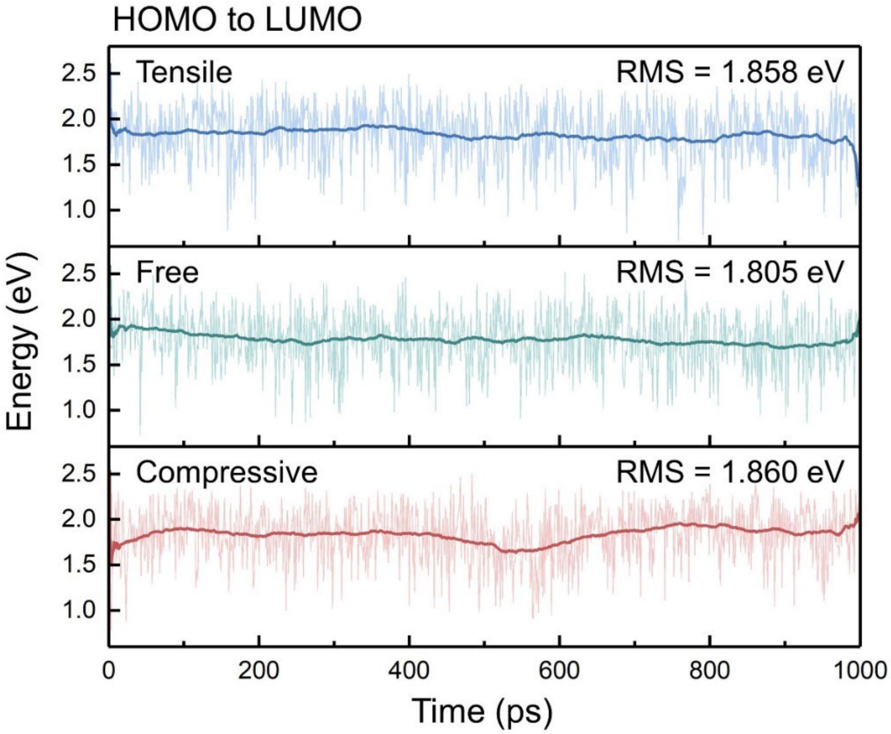

Figure S6. Evolution of the energy gap from HOMO to LUMO in the GB models. The thin lines are from DFT calculations with a timestep of 1 ps, and the thick lines indicate the moving average over 100 ps.

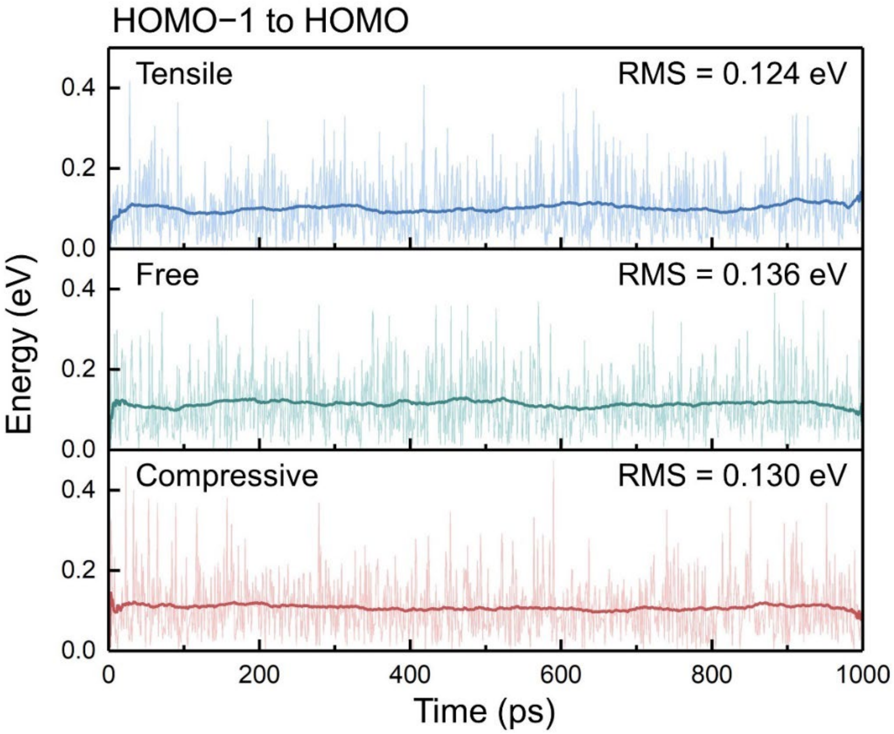

Figure S7. Evolution of the energy gap from HOMO−1 to HOMO in the GB models. The thin lines are from DFT calculations with a timestep of 1 ps, and the thick lines indicate the moving average over 100 ps.

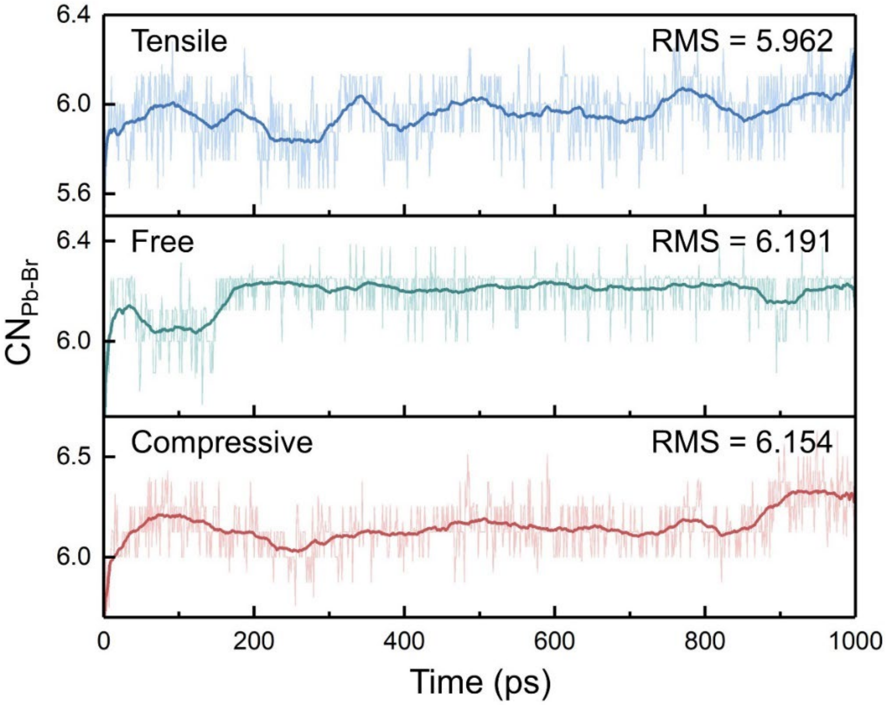

Figure S8. Evolution of the Pb−Br coordination number (CN_{Pb-Br}) in the GB models. The thin lines are from MLMD trajectories directly with a timestep of 0.5 ps, and the thick lines indicate the moving average over 50 ps.

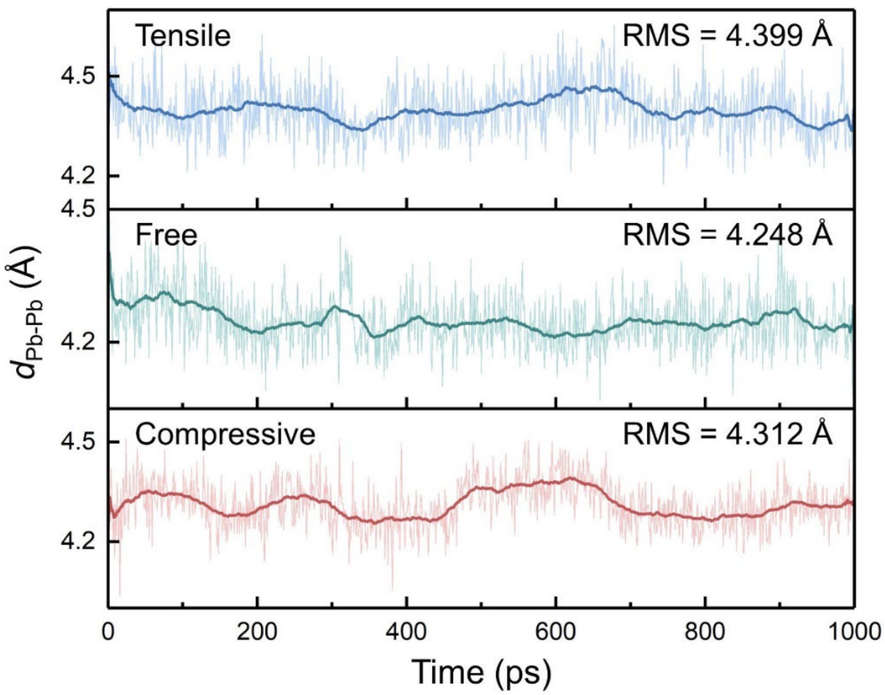

Figure S9. Evolution of the Pb−Pb distance (d_{Pb-Pb}) in the GB models. The thin lines are from MLMD trajectories directly with a timestep of 0.5 ps, and the thick lines indicate the moving average over 50 ps.

Table S1. Structural information of the tensile-strain model before sliding in .vasp format.

Tensile-strain model before sliding					
1.0					
11.8661	0.0000	0.0000			
0.0000	13.2668	0.0000			
0.0000	0.0000	53.0671			
Cs Pb	Br				
40 40	120				
Direct					
(before optimization)			(after optimization)		
0.2500	0.0000	0.0000	0.7500	0.9382	1.0000
0.7500	0.0000	0.0000	0.2500	0.9383	0.0000
0.2500	0.6000	0.0500	0.2500	0.5611	0.0505
0.7500	0.6000	0.0500	0.7501	0.5612	0.0504
0.2500	0.2000	0.1000	0.2500	0.2019	0.1104
0.7500	0.2000	0.1000	0.7500	0.2018	0.1104
0.2500	0.8000	0.1500	0.2500	0.8102	0.1483
0.7500	0.8000	0.1500	0.7500	0.8097	0.1483
0.2500	0.4000	0.2000	0.2500	0.4104	0.2008
0.7500	0.4000	0.2000	0.7500	0.4103	0.2008
0.2500	0.0000	0.2500	0.2500	0.0010	0.2501
0.7500	0.0000	0.2500	0.7500	0.0008	0.2501
0.2500	0.6000	0.3000	0.2500	0.5895	0.2992
0.7500	0.6000	0.3000	0.7500	0.5896	0.2992
0.2500	0.2000	0.3500	0.2500	0.1903	0.3517
0.7500	0.2000	0.3500	0.7499	0.1902	0.3517
0.2500	0.8000	0.4000	0.2500	0.7981	0.3895
0.7500	0.8000	0.4000	0.7500	0.7979	0.3897
0.2500	0.4000	0.4500	0.2501	0.4388	0.4496
0.7500	0.4000	0.4500	0.7500	0.4389	0.4496
0.2500	0.0000	0.5000	0.2501	0.0618	0.5000
0.7500	0.0000	0.5000	0.7500	0.0616	0.5000
0.2500	0.6000	0.9500	0.7500	0.5611	0.9495
0.7500	0.6000	0.9500	0.2499	0.5610	0.9495
0.2500	0.2000	0.9000	0.7500	0.2019	0.8895
0.7500	0.2000	0.9000	0.2500	0.2020	0.8896
0.2500	0.8000	0.8500	0.7500	0.8101	0.8517
0.7500	0.8000	0.8500	0.2500	0.8097	0.8517
0.2500	0.4000	0.8000	0.7500	0.4103	0.7992
0.7500	0.4000	0.8000	0.2500	0.4103	0.7992
0.2500	0.0000	0.7500	0.7500	0.0011	0.7498
0.7500	0.0000	0.7500	0.2500	0.0011	0.7498
0.2500	0.6000	0.7000	0.7500	0.5895	0.7008
0.7500	0.6000	0.7000	0.2500	0.5896	0.7009
0.2500	0.2000	0.6500	0.7500	0.1903	0.6483

0.7500	0.2000	0.6500	0.2500	0.1904	0.6483
0.2500	0.8000	0.6000	0.7500	0.7981	0.6105
0.7500	0.8000	0.6000	0.2500	0.7981	0.6104
0.2500	0.4000	0.5500	0.7500	0.4385	0.5505
0.7500	0.4000	0.5500	0.2500	0.4385	0.5505
0.5000	0.3000	0.0250	0.4999	0.2687	0.9666
0.0000	0.3000	0.0250	0.9997	0.2686	0.9666
0.5000	0.9000	0.0750	0.4999	0.8793	0.9249
0.0000	0.9000	0.0750	0.0000	0.8793	0.9249
0.0000	0.5000	0.1250	0.5000	0.5012	0.8707
0.5000	0.5000	0.1250	1.0000	0.5012	0.8707
0.0000	0.1000	0.1750	0.5000	0.0907	0.8251
0.5000	0.1000	0.1750	1.0000	0.0907	0.8251
0.5000	0.7000	0.2250	1.0000	0.7068	0.7736
0.0000	0.7000	0.2250	0.5001	0.7068	0.7736
0.5000	0.3000	0.2750	1.0000	0.2935	0.7265
0.0000	0.3000	0.2750	0.5000	0.2934	0.7265
0.0000	0.9000	0.3250	0.5000	0.9093	0.6750
0.5000	0.9000	0.3250	0.0000	0.9093	0.6750
0.0000	0.5000	0.3750	0.5000	0.4989	0.6293
0.5000	0.5000	0.3750	0.0001	0.4990	0.6293
0.0000	0.1000	0.4250	0.5000	0.1204	0.5751
0.5000	0.1000	0.4250	1.0000	0.1204	0.5751
0.0000	0.7000	0.4750	0.9997	0.7311	0.5334
0.5000	0.7000	0.4750	0.4998	0.7312	0.5334
0.0000	0.3000	0.9750	0.5001	0.2687	0.0334
0.5000	0.3000	0.9750	0.0002	0.2688	0.0334
0.0000	0.9000	0.9250	0.5000	0.8794	0.0751
0.5000	0.9000	0.9250	0.0000	0.8795	0.0751
0.0000	0.5000	0.8750	1.0000	0.5012	0.1293
0.5000	0.5000	0.8750	0.5000	0.5012	0.1293
0.0000	0.1000	0.8250	0.0000	0.0907	0.1749
0.5000	0.1000	0.8250	0.5000	0.0907	0.1749
0.5000	0.7000	0.7750	0.4999	0.7068	0.2265
0.0000	0.7000	0.7750	0.0001	0.7068	0.2265
0.5000	0.3000	0.7250	0.5000	0.2935	0.2735
0.0000	0.3000	0.7250	1.0000	0.2935	0.2735
0.0000	0.9000	0.6750	0.9999	0.9092	0.3250
0.5000	0.9000	0.6750	0.5001	0.9092	0.3251
0.0000	0.5000	0.6250	0.0000	0.4989	0.3708
0.5000	0.5000	0.6250	0.5000	0.4989	0.3708
0.0000	0.1000	0.5750	0.0000	0.1205	0.4248
0.5000	0.1000	0.5750	0.4999	0.1206	0.4249
0.5000	0.7000	0.5250	0.0002	0.7314	0.4666
0.0000	0.7000	0.5250	0.5000	0.7313	0.4666
0.2500	0.3000	0.0250	0.2499	0.2796	0.0442

0.7500	0.3000	0.0250	0.7499	0.2798	0.0441
0.2500	0.9000	0.0750	0.2500	0.9313	0.0815
0.7500	0.9000	0.0750	0.7500	0.9310	0.0817
0.2500	0.5000	0.1250	0.2500	0.4928	0.1168
0.7500	0.5000	0.1250	0.7500	0.4929	0.1167
0.2500	0.1000	0.1750	0.2500	0.1314	0.1833
0.7500	0.1000	0.1750	0.7500	0.1315	0.1833
0.2500	0.7000	0.2250	0.2499	0.6925	0.2149
0.7500	0.7000	0.2250	0.7500	0.6923	0.2151
0.2500	0.3000	0.2750	0.2500	0.3074	0.2850
0.7500	0.3000	0.2750	0.7500	0.3075	0.2851
0.2500	0.9000	0.3250	0.2500	0.8683	0.3167
0.7500	0.9000	0.3250	0.7500	0.8685	0.3167
0.2500	0.5000	0.3750	0.2500	0.5069	0.3833
0.7500	0.5000	0.3750	0.7500	0.5068	0.3833
0.2500	0.1000	0.4250	0.2500	0.0686	0.4184
0.7500	0.1000	0.4250	0.7500	0.0686	0.4185
0.2500	0.7000	0.4750	0.2500	0.7202	0.4557
0.7500	0.7000	0.4750	0.7500	0.7207	0.4560
0.5000	0.5000	0.0000	1.0000	0.4577	1.0000
0.0000	0.5000	0.0000	0.4999	0.4577	0.0000
0.0000	0.1000	0.0500	0.9997	0.0458	0.0364
0.5000	0.1000	0.0500	0.5004	0.0457	0.0364
0.5000	0.7000	0.1000	0.4998	0.7151	0.1140
0.0000	0.7000	0.1000	0.0002	0.7151	0.1139
0.5000	0.3000	0.1500	0.5000	0.3061	0.1581
0.0000	0.3000	0.1500	0.0001	0.3061	0.1581
0.0000	0.9000	0.2000	1.0000	0.8737	0.1889
0.5000	0.9000	0.2000	0.5000	0.8737	0.1889
0.0000	0.5000	0.2500	0.0001	0.5002	0.2501
0.5000	0.5000	0.2500	0.4999	0.5002	0.2501
0.0000	0.1000	0.3000	0.0000	0.1262	0.3111
0.5000	0.1000	0.3000	0.5000	0.1262	0.3111
0.5000	0.7000	0.3500	0.5001	0.6936	0.3418
0.0000	0.7000	0.3500	0.9998	0.6937	0.3418
0.5000	0.3000	0.4000	0.5000	0.2851	0.3861
0.0000	0.3000	0.4000	0.0000	0.2851	0.3861
0.5000	0.9000	0.4500	0.4999	0.9541	0.4636
0.0000	0.9000	0.4500	0.0002	0.9543	0.4635
0.5000	0.8000	0.0250	0.5000	0.7295	0.0359
0.0000	0.8000	0.0250	0.0001	0.7299	0.0359
0.0000	0.4000	0.0750	0.9998	0.3698	0.0799
0.5000	0.4000	0.0750	0.5000	0.3695	0.0799
0.0000	0.0000	0.1250	0.0003	0.0341	0.1217
0.5000	0.0000	0.1250	0.4998	0.0341	0.1216
0.5000	0.6000	0.1750	0.5005	0.5753	0.1799

0.0000	0.6000	0.1750	0.9994	0.5754	0.1799
0.5000	0.2000	0.2250	0.5000	0.1533	0.2306
0.0000	0.2000	0.2250	1.0000	0.1533	0.2306
0.5000	0.8000	0.2750	0.4999	0.8479	0.2693
0.0000	0.8000	0.2750	0.0001	0.8478	0.2692
0.5000	0.4000	0.3250	0.4999	0.4241	0.3202
0.0000	0.4000	0.3250	0.0002	0.4243	0.3202
0.0000	0.0000	0.3750	0.9998	0.9657	0.3783
0.5000	0.0000	0.3750	0.5002	0.9657	0.3783
0.0000	0.6000	0.4250	0.9995	0.6308	0.4200
0.5000	0.6000	0.4250	0.5003	0.6307	0.4200
0.5000	0.2000	0.4750	0.5000	0.2701	0.4641
0.0000	0.2000	0.4750	0.0002	0.2705	0.4640
0.2500	0.3000	0.9750	0.7501	0.2797	0.9557
0.7500	0.3000	0.9750	0.2501	0.2795	0.9559
0.2500	0.9000	0.9250	0.7499	0.9312	0.9185
0.7500	0.9000	0.9250	0.2500	0.9311	0.9184
0.2500	0.5000	0.8750	0.7501	0.4926	0.8832
0.7500	0.5000	0.8750	0.2501	0.4929	0.8832
0.2500	0.1000	0.8250	0.7500	0.1314	0.8167
0.7500	0.1000	0.8250	0.2500	0.1314	0.8167
0.2500	0.7000	0.7750	0.7500	0.6924	0.7851
0.7500	0.7000	0.7750	0.2500	0.6923	0.7849
0.2500	0.3000	0.7250	0.7500	0.3073	0.7150
0.7500	0.3000	0.7250	0.2500	0.3072	0.7150
0.2500	0.9000	0.6750	0.7500	0.8685	0.6833
0.7500	0.9000	0.6750	0.2500	0.8685	0.6834
0.2500	0.5000	0.6250	0.7501	0.5076	0.6169
0.7500	0.5000	0.6250	0.2501	0.5073	0.6168
0.2500	0.1000	0.5750	0.7500	0.0685	0.5816
0.7500	0.1000	0.5750	0.2500	0.0687	0.5816
0.2500	0.7000	0.5250	0.7502	0.7195	0.5442
0.7500	0.7000	0.5250	0.2502	0.7196	0.5441
0.5000	0.5000	0.5000	0.4998	0.5421	0.5001
0.0000	0.5000	0.5000	0.0001	0.5421	0.5000
0.5000	0.1000	0.9500	0.0001	0.0456	0.9636
0.0000	0.1000	0.9500	0.4999	0.0457	0.9636
0.5000	0.7000	0.9000	0.9999	0.7150	0.8861
0.0000	0.7000	0.9000	0.5001	0.7150	0.8861
0.5000	0.3000	0.8500	0.0000	0.3060	0.8419
0.0000	0.3000	0.8500	0.5000	0.3060	0.8419
0.5000	0.9000	0.8000	0.0001	0.8737	0.8111
0.0000	0.9000	0.8000	0.4999	0.8736	0.8112
0.0000	0.5000	0.7500	0.5000	0.5002	0.7499
0.5000	0.5000	0.7500	0.9999	0.5001	0.7499
0.5000	0.1000	0.7000	1.0000	0.1263	0.6889

0.0000	0.1000	0.7000	0.5000	0.1262	0.6889
0.5000	0.7000	0.6500	0.0000	0.6935	0.6583
0.0000	0.7000	0.6500	0.4999	0.6935	0.6583
0.5000	0.3000	0.6000	0.9997	0.2853	0.6137
0.0000	0.3000	0.6000	0.5001	0.2853	0.6137
0.0000	0.9000	0.5500	0.5001	0.9542	0.5363
0.5000	0.9000	0.5500	0.0000	0.9542	0.5363
0.0000	0.8000	0.9750	0.4999	0.7293	0.9640
0.5000	0.8000	0.9750	0.0001	0.7293	0.9640
0.0000	0.4000	0.9250	0.4999	0.3695	0.9201
0.5000	0.4000	0.9250	0.0004	0.3693	0.9200
0.0000	0.0000	0.8750	0.4999	0.0340	0.8784
0.5000	0.0000	0.8750	1.0000	0.0342	0.8784
0.5000	0.6000	0.8250	0.0002	0.5754	0.8201
0.0000	0.6000	0.8250	0.4996	0.5753	0.8201
0.5000	0.2000	0.7750	0.9999	0.1531	0.7694
0.0000	0.2000	0.7750	0.5000	0.1531	0.7694
0.5000	0.8000	0.7250	1.0000	0.8480	0.7308
0.0000	0.8000	0.7250	0.5000	0.8481	0.7308
0.0000	0.4000	0.6750	0.4999	0.4239	0.6798
0.5000	0.4000	0.6750	0.0001	0.4239	0.6798
0.0000	0.0000	0.6250	0.4999	0.9657	0.6217
0.5000	0.0000	0.6250	0.0000	0.9658	0.6217
0.0000	0.6000	0.5750	0.5002	0.6312	0.5801
0.5000	0.6000	0.5750	0.0003	0.6309	0.5800
0.0000	0.2000	0.5250	0.5000	0.2696	0.5357
0.5000	0.2000	0.5250	0.9999	0.2696	0.5357

Table S2. Structural information of the tensile-strain model after sliding in .vasp format.

Tensile-strain model after sliding					
1.0					
11.8661	0.0000	0.0000			
0.0000	13.2668	0.0000			
0.0000	0.0000	53.0671			
Cs Pb	Br				
40 40	120				
Direct					
(before optimization)			(after optimization)		
0.2500	0.0000	0.0000	0.2500	0.9736	0.0089
0.7500	0.0000	0.0000	0.7500	0.9736	0.0089
0.2500	0.6000	0.0500	0.2501	0.6257	0.0475
0.7500	0.6000	0.0500	0.7500	0.6255	0.0475
0.2500	0.2000	0.1000	0.2500	0.1806	0.1037

0.7500	0.2000	0.1000	0.7500	0.1806	0.1037
0.2500	0.8000	0.1500	0.2500	0.8026	0.1502
0.7500	0.8000	0.1500	0.7500	0.8026	0.1502
0.2500	0.4000	0.2000	0.2500	0.3809	0.2043
0.7500	0.4000	0.2000	0.7500	0.3809	0.2043
0.2500	0.0000	0.2500	0.2500	0.0022	0.2497
0.7500	0.0000	0.2500	0.7500	0.0023	0.2497
0.2500	0.6000	0.3000	0.2500	0.6061	0.2938
0.7500	0.6000	0.3000	0.7500	0.6061	0.2938
0.2500	0.2000	0.3500	0.2501	0.1721	0.3466
0.7500	0.2000	0.3500	0.7500	0.1722	0.3466
0.2500	0.8000	0.4000	0.2500	0.8211	0.4001
0.7500	0.8000	0.4000	0.7500	0.8211	0.4001
0.2500	0.4000	0.4500	0.2500	0.3818	0.4457
0.7500	0.4000	0.4500	0.7500	0.3818	0.4457
0.0000	0.0000	0.5000	0.0000	0.0268	0.5089
0.5000	0.0000	0.5000	0.5000	0.0268	0.5089
0.0000	0.6000	0.9500	1.0000	0.6183	0.9458
0.5000	0.6000	0.9500	0.5000	0.6183	0.9458
0.0000	0.2000	0.9000	1.0000	0.1790	0.9000
0.5000	0.2000	0.9000	0.5000	0.1790	0.9000
0.0000	0.8000	0.8500	0.0000	0.8279	0.8467
0.5000	0.8000	0.8500	0.5000	0.8279	0.8467
0.0000	0.4000	0.8000	0.0000	0.3937	0.7939
0.5000	0.4000	0.8000	0.5000	0.3937	0.7939
0.0000	0.0000	0.7500	0.0001	0.9979	0.7498
0.5000	0.0000	0.7500	0.5001	0.9979	0.7498
0.0000	0.6000	0.7000	1.0000	0.6188	0.7044
0.5000	0.6000	0.7000	0.5000	0.6188	0.7044
0.0000	0.2000	0.6500	1.0000	0.1955	0.6504
0.5000	0.2000	0.6500	0.5001	0.1956	0.6504
0.0000	0.8000	0.6000	0.0001	0.8194	0.6038
0.5000	0.8000	0.6000	0.5000	0.8194	0.6038
0.0000	0.4000	0.5500	0.0000	0.3745	0.5474
0.5000	0.4000	0.5500	0.5000	0.3745	0.5474
0.5000	0.3000	0.0250	0.7499	0.3033	0.9716
0.0000	0.3000	0.0250	0.2500	0.3033	0.9716
0.5000	0.9000	0.0750	0.7500	0.8847	0.9245
0.0000	0.9000	0.0750	0.2500	0.8846	0.9245
0.0000	0.5000	0.1250	0.7500	0.4976	0.8739
0.5000	0.5000	0.1250	0.2500	0.4975	0.8739
0.0000	0.1000	0.1750	0.7500	0.1079	0.8258
0.5000	0.1000	0.1750	0.2500	0.1079	0.8258
0.5000	0.7000	0.2250	0.2500	0.6966	0.7765
0.0000	0.7000	0.2250	0.7500	0.6966	0.7765
0.5000	0.3000	0.2750	0.2500	0.3034	0.7227

0.0000	0.3000	0.2750	0.7500	0.3034	0.7227
0.0000	0.9000	0.3250	0.7500	0.8951	0.6751
0.5000	0.9000	0.3250	0.2500	0.8951	0.6751
0.0000	0.5000	0.3750	0.7500	0.5026	0.6246
0.5000	0.5000	0.3750	0.2500	0.5026	0.6246
0.0000	0.1000	0.4250	0.7500	0.1103	0.5717
0.5000	0.1000	0.4250	0.2500	0.1103	0.5717
0.0000	0.7000	0.4750	0.2499	0.6942	0.5315
0.5000	0.7000	0.4750	0.7499	0.6942	0.5315
0.7500	0.3000	0.9750	0.5000	0.3067	0.0314
0.2500	0.3000	0.9750	0.0000	0.3067	0.0314
0.7500	0.9000	0.9250	0.5000	0.8896	0.0715
0.2500	0.9000	0.9250	1.0000	0.8896	0.0715
0.7500	0.5000	0.8750	0.0001	0.4971	0.1246
0.2500	0.5000	0.8750	0.5000	0.4972	0.1246
0.7500	0.1000	0.8250	0.0000	0.1040	0.1751
0.2500	0.1000	0.8250	0.5000	0.1040	0.1751
0.2500	0.7000	0.7750	0.5000	0.6963	0.2228
0.7500	0.7000	0.7750	0.0000	0.6962	0.2228
0.2500	0.3000	0.7250	0.5000	0.3033	0.2764
0.7500	0.3000	0.7250	0.0000	0.3033	0.2764
0.7500	0.9000	0.6750	0.0001	0.8918	0.3258
0.2500	0.9000	0.6750	0.5001	0.8918	0.3258
0.7500	0.5000	0.6250	0.0000	0.5030	0.3739
0.2500	0.5000	0.6250	0.5000	0.5030	0.3739
0.7500	0.1000	0.5750	0.0000	0.1156	0.4244
0.2500	0.1000	0.5750	0.5000	0.1156	0.4244
0.2500	0.7000	0.5250	0.9993	0.6967	0.4716
0.7500	0.7000	0.5250	0.4993	0.6967	0.4715
0.2500	0.3000	0.0250	0.2500	0.2478	0.0287
0.7500	0.3000	0.0250	0.7500	0.2479	0.0287
0.2500	0.9000	0.0750	0.2500	0.9041	0.0837
0.7500	0.9000	0.0750	0.7500	0.9042	0.0837
0.2500	0.5000	0.1250	0.2500	0.4846	0.1107
0.7500	0.5000	0.1250	0.7500	0.4842	0.1107
0.2500	0.1000	0.1750	0.2500	0.1089	0.1854
0.7500	0.1000	0.1750	0.7500	0.1090	0.1854
0.2500	0.7000	0.2250	0.2500	0.6554	0.2164
0.7500	0.7000	0.2250	0.7500	0.6553	0.2164
0.2500	0.3000	0.2750	0.2500	0.3243	0.2875
0.7500	0.3000	0.2750	0.7500	0.3243	0.2875
0.2500	0.9000	0.3250	0.2500	0.8922	0.3134
0.7500	0.9000	0.3250	0.7500	0.8921	0.3134
0.2500	0.5000	0.3750	0.2499	0.5379	0.3855
0.7500	0.5000	0.3750	0.7499	0.5379	0.3855
0.2500	0.1000	0.4250	0.2501	0.1085	0.4130

0.7500	0.1000	0.4250	0.7501	0.1085	0.4130
0.2500	0.7000	0.4750	0.2498	0.7604	0.4745
0.7500	0.7000	0.4750	0.7498	0.7603	0.4745
0.5000	0.5000	0.0000	0.5002	0.5116	0.0128
0.0000	0.5000	0.0000	0.0002	0.5116	0.0128
0.0000	0.1000	0.0500	1.0000	0.0795	0.0419
0.5000	0.1000	0.0500	0.5000	0.0795	0.0419
0.5000	0.7000	0.1000	0.5003	0.6770	0.0913
0.0000	0.7000	0.1000	0.9999	0.6770	0.0913
0.5000	0.3000	0.1500	0.4998	0.3228	0.1619
0.0000	0.3000	0.1500	0.0003	0.3228	0.1619
0.0000	0.9000	0.2000	0.0001	0.9080	0.2044
0.5000	0.9000	0.2000	0.5000	0.9080	0.2044
0.0000	0.5000	0.2500	1.0000	0.4799	0.2412
0.5000	0.5000	0.2500	0.5000	0.4799	0.2412
0.0000	0.1000	0.3000	0.0000	0.1010	0.3029
0.5000	0.1000	0.3000	0.5000	0.1010	0.3029
0.5000	0.7000	0.3500	0.5000	0.7210	0.3629
0.0000	0.7000	0.3500	0.0000	0.7210	0.3629
0.5000	0.3000	0.4000	0.5001	0.2802	0.3873
0.0000	0.3000	0.4000	0.0001	0.2802	0.3873
0.5000	0.9000	0.4500	0.5000	0.8848	0.4455
0.0000	0.9000	0.4500	0.9999	0.8847	0.4455
0.5000	0.8000	0.0250	0.5000	0.8010	0.0224
0.0000	0.8000	0.0250	0.0000	0.8010	0.0224
0.0000	0.4000	0.0750	0.0001	0.3280	0.0862
0.5000	0.4000	0.0750	0.5000	0.3280	0.0862
0.0000	0.0000	0.1250	0.0000	0.0084	0.1250
0.5000	0.0000	0.1250	0.5000	0.0083	0.1250
0.5000	0.6000	0.1750	0.5000	0.6446	0.1675
0.0000	0.6000	0.1750	1.0000	0.6446	0.1675
0.5000	0.2000	0.2250	0.4999	0.1827	0.2298
0.0000	0.2000	0.2250	0.0001	0.1827	0.2298
0.5000	0.8000	0.2750	0.5000	0.7732	0.2759
0.0000	0.8000	0.2750	0.0001	0.7732	0.2759
0.5000	0.4000	0.3250	0.5000	0.4612	0.3193
0.0000	0.4000	0.3250	0.0000	0.4612	0.3193
0.0000	0.0000	0.3750	0.0001	0.9979	0.3740
0.5000	0.0000	0.3750	0.5001	0.9979	0.3740
0.0000	0.6000	0.4250	0.9999	0.5480	0.4323
0.5000	0.6000	0.4250	0.4999	0.5480	0.4323
0.5000	0.2000	0.4750	0.5000	0.2471	0.4679
0.0000	0.2000	0.4750	1.0000	0.2470	0.4679
0.0000	0.3000	0.9750	0.9999	0.2399	0.9742
0.5000	0.3000	0.9750	0.4999	0.2399	0.9742
0.0000	0.9000	0.9250	0.0000	0.8923	0.9131

0.5000	0.9000	0.9250	0.5000	0.8923	0.9131
0.0000	0.5000	0.8750	1.0000	0.4630	0.8855
0.5000	0.5000	0.8750	0.5000	0.4630	0.8855
0.0000	0.1000	0.8250	1.0000	0.1066	0.8136
0.5000	0.1000	0.8250	0.5000	0.1067	0.8136
0.0000	0.7000	0.7750	0.0000	0.6760	0.7876
0.5000	0.7000	0.7750	0.5000	0.6760	0.7876
0.0000	0.3000	0.7250	0.0000	0.3432	0.7159
0.5000	0.3000	0.7250	0.5000	0.3432	0.7160
0.0000	0.9000	0.6750	0.0000	0.8926	0.6855
0.5000	0.9000	0.6750	0.5000	0.8925	0.6855
0.0000	0.5000	0.6250	0.0000	0.5169	0.6106
0.5000	0.5000	0.6250	0.5000	0.5169	0.6106
0.0000	0.1000	0.5750	0.0001	0.0941	0.5839
0.5000	0.1000	0.5750	0.5001	0.0943	0.5839
0.0000	0.7000	0.5250	0.9999	0.7525	0.5287
0.5000	0.7000	0.5250	0.4999	0.7525	0.5286
0.2500	0.5000	0.5000	0.2501	0.4892	0.5127
0.7500	0.5000	0.5000	0.7502	0.4892	0.5127
0.2500	0.1000	0.9500	0.2499	0.1153	0.9455
0.7500	0.1000	0.9500	0.7499	0.1153	0.9455
0.2500	0.7000	0.9000	0.2500	0.7204	0.8873
0.7500	0.7000	0.9000	0.7500	0.7204	0.8873
0.2500	0.3000	0.8500	0.2500	0.2797	0.8627
0.7500	0.3000	0.8500	0.7500	0.2797	0.8627
0.2500	0.9000	0.8000	0.2501	0.8986	0.8030
0.7500	0.9000	0.8000	0.7500	0.8986	0.8030
0.7500	0.5000	0.7500	0.7500	0.5193	0.7414
0.2500	0.5000	0.7500	0.2500	0.5193	0.7414
0.2500	0.1000	0.7000	0.2501	0.0915	0.7044
0.7500	0.1000	0.7000	0.7500	0.0915	0.7043
0.2500	0.7000	0.6500	0.2500	0.6759	0.6621
0.7500	0.7000	0.6500	0.7499	0.6758	0.6621
0.2500	0.3000	0.6000	0.2501	0.3230	0.5913
0.7500	0.3000	0.6000	0.7501	0.3230	0.5913
0.7500	0.9000	0.5500	0.7500	0.9211	0.5420
0.2500	0.9000	0.5500	0.2500	0.9211	0.5420
0.7500	0.8000	0.9750	0.7500	0.7533	0.9680
0.2500	0.8000	0.9750	0.2500	0.7533	0.9680
0.7500	0.4000	0.9250	0.7500	0.4522	0.9323
0.2500	0.4000	0.9250	0.2500	0.4522	0.9323
0.7500	0.0000	0.8750	0.7500	0.0031	0.8742
0.2500	0.0000	0.8750	0.2500	0.0031	0.8742
0.2500	0.6000	0.8250	0.2500	0.5395	0.8194
0.7500	0.6000	0.8250	0.7500	0.5395	0.8194
0.2500	0.2000	0.7750	0.2500	0.2261	0.7758

0.7500	0.2000	0.7750	0.7500	0.2261	0.7758
0.2500	0.8000	0.7250	0.2500	0.8162	0.7298
0.7500	0.8000	0.7250	0.7500	0.8162	0.7298
0.7500	0.4000	0.6750	0.7500	0.3542	0.6673
0.2500	0.4000	0.6750	0.2500	0.3541	0.6673
0.7500	0.0000	0.6250	0.7500	0.9924	0.6253
0.2500	0.0000	0.6250	0.2500	0.9925	0.6253
0.7500	0.6000	0.5750	0.7500	0.6726	0.5863
0.2500	0.6000	0.5750	0.2500	0.6726	0.5863
0.7500	0.2000	0.5250	0.7500	0.1998	0.5226
0.2500	0.2000	0.5250	0.2500	0.1998	0.5226

Table S3. Structural information of the strain-free model after sliding in .vasp format.

Strain-free model after sliding					
1.0					
11.8661	0.0000	0.0000			
0.0000	13.2668	0.0000			
0.0000	0.0000	52.0058			
Cs Pb	Br				
40 40	120				
Direct					
(before optimization)			(after optimization)		
0.2500	0.0000	0.0000	0.2500	0.9724	0.0095
0.7500	0.0000	0.0000	0.7500	0.9723	0.0095
0.2500	0.6000	0.0500	0.2500	0.6204	0.0423
0.7500	0.6000	0.0500	0.7502	0.6202	0.0423
0.2500	0.2000	0.1000	0.2500	0.1772	0.1036
0.7500	0.2000	0.1000	0.7500	0.1772	0.1036
0.2500	0.8000	0.1500	0.2500	0.8225	0.1525
0.7500	0.8000	0.1500	0.7500	0.8225	0.1525
0.2500	0.4000	0.2000	0.2501	0.3754	0.2035
0.7500	0.4000	0.2000	0.7500	0.3754	0.2035
0.2500	0.0000	0.2500	0.2500	0.0171	0.2521
0.7500	0.0000	0.2500	0.7500	0.0171	0.2521
0.2500	0.6000	0.3000	0.2500	0.6070	0.2935
0.7500	0.6000	0.3000	0.7500	0.6070	0.2935
0.2500	0.2000	0.3500	0.2500	0.1699	0.3473
0.7500	0.2000	0.3500	0.7501	0.1699	0.3473
0.2500	0.8000	0.4000	0.2500	0.8297	0.4001
0.7500	0.8000	0.4000	0.7500	0.8297	0.4001
0.2500	0.4000	0.4500	0.2500	0.3824	0.4448
0.7500	0.4000	0.4500	0.7500	0.3822	0.4447
0.0000	0.0000	0.5000	0.9999	0.0273	0.5099

0.5000	0.0000	0.5000	0.5000	0.0273	0.5099
0.0000	0.6000	0.9500	0.0001	0.6172	0.9446
0.5000	0.6000	0.9500	0.5000	0.6173	0.9446
0.0000	0.2000	0.9000	1.0000	0.1739	0.9004
0.5000	0.2000	0.9000	0.4999	0.1744	0.9005
0.0000	0.8000	0.8500	0.0000	0.8318	0.8476
0.5000	0.8000	0.8500	0.5000	0.8317	0.8476
0.0000	0.4000	0.8000	1.0000	0.3922	0.7941
0.5000	0.4000	0.8000	0.5001	0.3920	0.7939
0.0000	0.0000	0.7500	0.0000	0.9842	0.7520
0.5000	0.0000	0.7500	0.4999	0.9836	0.7518
0.0000	0.6000	0.7000	1.0000	0.6236	0.7039
0.5000	0.6000	0.7000	0.5000	0.6235	0.7039
0.0000	0.2000	0.6500	1.0000	0.1775	0.6533
0.5000	0.2000	0.6500	0.4999	0.1772	0.6533
0.0000	0.8000	0.6000	0.9999	0.8244	0.6025
0.5000	0.8000	0.6000	0.5000	0.8241	0.6025
0.0000	0.4000	0.5500	1.0000	0.3784	0.5428
0.5000	0.4000	0.5500	0.5000	0.3783	0.5428
0.5000	0.3000	0.0250	0.7498	0.3000	0.9723
0.0000	0.3000	0.0250	0.2496	0.3001	0.9723
0.5000	0.9000	0.0750	0.7501	0.8798	0.9262
0.0000	0.9000	0.0750	0.2501	0.8800	0.9262
0.0000	0.5000	0.1250	0.7501	0.4949	0.8746
0.5000	0.5000	0.1250	0.2499	0.4950	0.8746
0.0000	0.1000	0.1750	0.7499	0.1077	0.8263
0.5000	0.1000	0.1750	0.2501	0.1078	0.8263
0.5000	0.7000	0.2250	0.2500	0.6911	0.7769
0.0000	0.7000	0.2250	0.7500	0.6911	0.7769
0.5000	0.3000	0.2750	0.2499	0.3000	0.7220
0.0000	0.3000	0.2750	0.7500	0.3000	0.7220
0.0000	0.9000	0.3250	0.7501	0.8929	0.6749
0.5000	0.9000	0.3250	0.2498	0.8929	0.6749
0.0000	0.5000	0.3750	0.7499	0.4987	0.6247
0.5000	0.5000	0.3750	0.2501	0.4987	0.6247
0.0000	0.1000	0.4250	0.7499	0.1223	0.5716
0.5000	0.1000	0.4250	0.2500	0.1222	0.5716
0.0000	0.7000	0.4750	0.2499	0.6975	0.5317
0.5000	0.7000	0.4750	0.7500	0.6974	0.5317
0.7500	0.3000	0.9750	0.5000	0.3004	0.0316
0.2500	0.3000	0.9750	0.0001	0.3003	0.0316
0.7500	0.9000	0.9250	0.5000	0.8773	0.0711
0.2500	0.9000	0.9250	1.0000	0.8772	0.0711
0.7500	0.5000	0.8750	0.9999	0.5002	0.1242
0.2500	0.5000	0.8750	0.4999	0.5002	0.1242
0.7500	0.1000	0.8250	0.0000	0.1049	0.1748

0.2500	0.1000	0.8250	0.5000	0.1050	0.1748
0.2500	0.7000	0.7750	0.5000	0.7000	0.2217
0.7500	0.7000	0.7750	0.0000	0.7000	0.2217
0.2500	0.3000	0.7250	0.5001	0.3092	0.2765
0.7500	0.3000	0.7250	0.0000	0.3093	0.2765
0.7500	0.9000	0.6750	0.0004	0.8920	0.3260
0.2500	0.9000	0.6750	0.5004	0.8920	0.3261
0.7500	0.5000	0.6250	1.0000	0.5068	0.3745
0.2500	0.5000	0.6250	0.5000	0.5068	0.3745
0.7500	0.1000	0.5750	0.0000	0.1205	0.4259
0.2500	0.1000	0.5750	0.5000	0.1206	0.4259
0.2500	0.7000	0.5250	0.9999	0.7005	0.4719
0.7500	0.7000	0.5250	0.5000	0.7005	0.4719
0.2500	0.3000	0.0250	0.2500	0.2431	0.0302
0.7500	0.3000	0.0250	0.7500	0.2431	0.0302
0.2500	0.9000	0.0750	0.2499	0.9048	0.0824
0.7500	0.9000	0.0750	0.7500	0.9050	0.0824
0.2500	0.5000	0.1250	0.2499	0.4731	0.1111
0.7500	0.5000	0.1250	0.7500	0.4731	0.1111
0.2500	0.1000	0.1750	0.2500	0.0831	0.1834
0.7500	0.1000	0.1750	0.7500	0.0830	0.1833
0.2500	0.7000	0.2250	0.2500	0.6521	0.2164
0.7500	0.7000	0.2250	0.7500	0.6521	0.2164
0.2500	0.3000	0.2750	0.2500	0.3190	0.2879
0.7500	0.3000	0.2750	0.7500	0.3193	0.2880
0.2500	0.9000	0.3250	0.2502	0.8976	0.3149
0.7500	0.9000	0.3250	0.7502	0.8974	0.3149
0.2500	0.5000	0.3750	0.2501	0.5459	0.3849
0.7500	0.5000	0.3750	0.7501	0.5459	0.3848
0.2500	0.1000	0.4250	0.2500	0.1126	0.4147
0.7500	0.1000	0.4250	0.7500	0.1126	0.4148
0.2500	0.7000	0.4750	0.2500	0.7661	0.4736
0.7500	0.7000	0.4750	0.7500	0.7666	0.4736
0.5000	0.5000	0.0000	0.5001	0.5024	0.0100
0.0000	0.5000	0.0000	0.0005	0.5022	0.0100
0.0000	0.1000	0.0500	0.9999	0.0753	0.0443
0.5000	0.1000	0.0500	0.5000	0.0754	0.0443
0.5000	0.7000	0.1000	0.5001	0.6576	0.0846
0.0000	0.7000	0.1000	1.0000	0.6575	0.0846
0.5000	0.3000	0.1500	0.5001	0.3232	0.1615
0.0000	0.3000	0.1500	0.0000	0.3231	0.1615
0.0000	0.9000	0.2000	0.0000	0.9170	0.2085
0.5000	0.9000	0.2000	0.5000	0.9170	0.2085
0.0000	0.5000	0.2500	0.0000	0.4809	0.2396
0.5000	0.5000	0.2500	0.5000	0.4808	0.2396
0.0000	0.1000	0.3000	0.0000	0.1006	0.3015

0.5000	0.1000	0.3000	0.5002	0.1005	0.3015
0.5000	0.7000	0.3500	0.5002	0.7252	0.3647
0.0000	0.7000	0.3500	0.0000	0.7252	0.3647
0.5000	0.3000	0.4000	0.5001	0.2839	0.3878
0.0000	0.3000	0.4000	0.9999	0.2838	0.3878
0.5000	0.9000	0.4500	0.4998	0.8894	0.4456
0.0000	0.9000	0.4500	0.0001	0.8893	0.4456
0.5000	0.8000	0.0250	0.5000	0.8016	0.0197
0.0000	0.8000	0.0250	1.0000	0.8016	0.0197
0.0000	0.4000	0.0750	0.9999	0.3249	0.0869
0.5000	0.4000	0.0750	0.4999	0.3248	0.0869
0.0000	0.0000	0.1250	0.0001	0.9960	0.1256
0.5000	0.0000	0.1250	0.4998	0.9957	0.1257
0.5000	0.6000	0.1750	0.5001	0.6640	0.1646
0.0000	0.6000	0.1750	1.0000	0.6640	0.1646
0.5000	0.2000	0.2250	0.5001	0.1901	0.2289
0.0000	0.2000	0.2250	0.9999	0.1900	0.2289
0.5000	0.8000	0.2750	0.5000	0.7675	0.2770
0.0000	0.8000	0.2750	1.0000	0.7675	0.2770
0.5000	0.4000	0.3250	0.4999	0.4682	0.3187
0.0000	0.4000	0.3250	0.0000	0.4682	0.3187
0.0000	0.0000	0.3750	0.9999	0.0037	0.3746
0.5000	0.0000	0.3750	0.5002	0.0038	0.3746
0.0000	0.6000	0.4250	0.9999	0.5467	0.4333
0.5000	0.6000	0.4250	0.5002	0.5467	0.4333
0.5000	0.2000	0.4750	0.5000	0.2550	0.4697
0.0000	0.2000	0.4750	0.0000	0.2550	0.4696
0.0000	0.3000	0.9750	0.0000	0.2328	0.9736
0.5000	0.3000	0.9750	0.5000	0.2332	0.9736
0.0000	0.9000	0.9250	0.0000	0.8926	0.9151
0.5000	0.9000	0.9250	0.5000	0.8923	0.9151
0.0000	0.5000	0.8750	0.0000	0.4555	0.8849
0.5000	0.5000	0.8750	0.5000	0.4562	0.8848
0.0000	0.1000	0.8250	1.0000	0.1015	0.8151
0.5000	0.1000	0.8250	0.5000	0.1011	0.8149
0.0000	0.7000	0.7750	0.0000	0.6804	0.7887
0.5000	0.7000	0.7750	0.5000	0.6802	0.7887
0.0000	0.3000	0.7250	0.9999	0.3480	0.7169
0.5000	0.3000	0.7250	0.4999	0.3480	0.7171
0.0000	0.9000	0.6750	1.0000	0.9188	0.6833
0.5000	0.9000	0.6750	0.4999	0.9180	0.6830
0.0000	0.5000	0.6250	0.9999	0.5277	0.6121
0.5000	0.5000	0.6250	0.5000	0.5280	0.6119
0.0000	0.1000	0.5750	0.9999	0.0988	0.5832
0.5000	0.1000	0.5750	0.4999	0.0982	0.5832
0.0000	0.7000	0.5250	1.0000	0.7553	0.5300

0.5000	0.7000	0.5250	0.5000	0.7551	0.5300
0.2500	0.5000	0.5000	0.2499	0.4957	0.5102
0.7500	0.5000	0.5000	0.7502	0.4957	0.5102
0.2500	0.1000	0.9500	0.2500	0.1124	0.9456
0.7500	0.1000	0.9500	0.7500	0.1123	0.9456
0.2500	0.7000	0.9000	0.2499	0.7182	0.8877
0.7500	0.7000	0.9000	0.7502	0.7182	0.8877
0.2500	0.3000	0.8500	0.2501	0.2764	0.8645
0.7500	0.3000	0.8500	0.7499	0.2763	0.8645
0.2500	0.9000	0.8000	0.2500	0.8989	0.8022
0.7500	0.9000	0.8000	0.7500	0.8989	0.8022
0.7500	0.5000	0.7500	0.7501	0.5199	0.7397
0.2500	0.5000	0.7500	0.2498	0.5199	0.7397
0.2500	0.1000	0.7000	0.2502	0.0832	0.7084
0.7500	0.1000	0.7000	0.7497	0.0832	0.7084
0.2500	0.7000	0.6500	0.2500	0.6740	0.6625
0.7500	0.7000	0.6500	0.7499	0.6740	0.6624
0.2500	0.3000	0.6000	0.2500	0.3420	0.5849
0.7500	0.3000	0.6000	0.7500	0.3419	0.5849
0.7500	0.9000	0.5500	0.7499	0.9231	0.5448
0.2500	0.9000	0.5500	0.2500	0.9232	0.5448
0.7500	0.8000	0.9750	0.7500	0.7434	0.9696
0.2500	0.8000	0.9750	0.2500	0.7435	0.9696
0.7500	0.4000	0.9250	0.7500	0.4529	0.9333
0.2500	0.4000	0.9250	0.2501	0.4529	0.9333
0.7500	0.0000	0.8750	0.7498	0.9978	0.8750
0.2500	0.0000	0.8750	0.2502	0.9977	0.8750
0.2500	0.6000	0.8250	0.2500	0.5309	0.8188
0.7500	0.6000	0.8250	0.7501	0.5309	0.8188
0.2500	0.2000	0.7750	0.2498	0.2330	0.7771
0.7500	0.2000	0.7750	0.7503	0.2329	0.7771
0.2500	0.8000	0.7250	0.2501	0.8114	0.7294
0.7500	0.8000	0.7250	0.7498	0.8114	0.7295
0.7500	0.4000	0.6750	0.7498	0.3333	0.6647
0.2500	0.4000	0.6750	0.2501	0.3333	0.6647
0.7500	0.0000	0.6250	0.7501	0.0010	0.6257
0.2500	0.0000	0.6250	0.2497	0.0008	0.6257
0.7500	0.6000	0.5750	0.7502	0.6731	0.5871
0.2500	0.6000	0.5750	0.2497	0.6732	0.5871
0.7500	0.2000	0.5250	0.7500	0.1975	0.5202
0.2500	0.2000	0.5250	0.2500	0.1975	0.5202

Table S4. Structural information of the compressive-strain model after sliding in .vasp format.

Compressive-strain model after sliding					
1.0					
11.8661	0.0000	0.0000			
0.0000	13.2668	0.0000			
0.0000	0.0000	50.9444			
Cs Pb	Br				
40 40	120				
Direct					
(before optimization)			(after optimization)		
0.2500	0.0000	0.0000	0.2501	0.9719	0.0092
0.7500	0.0000	0.0000	0.7500	0.9719	0.0092
0.2500	0.6000	0.0500	0.2500	0.6221	0.0412
0.7500	0.6000	0.0500	0.7500	0.6221	0.0412
0.2500	0.2000	0.1000	0.2499	0.1704	0.1035
0.7500	0.2000	0.1000	0.7500	0.1704	0.1035
0.2500	0.8000	0.1500	0.2500	0.8212	0.1525
0.7500	0.8000	0.1500	0.7500	0.8212	0.1525
0.2500	0.4000	0.2000	0.2500	0.3743	0.2042
0.7500	0.4000	0.2000	0.7500	0.3743	0.2042
0.2500	0.0000	0.2500	0.2499	0.0102	0.2516
0.7500	0.0000	0.2500	0.7500	0.0102	0.2516
0.2500	0.6000	0.3000	0.2500	0.6094	0.2957
0.7500	0.6000	0.3000	0.7500	0.6094	0.2957
0.2500	0.2000	0.3500	0.2500	0.1700	0.3474
0.7500	0.2000	0.3500	0.7500	0.1700	0.3474
0.2500	0.8000	0.4000	0.2500	0.8307	0.4006
0.7500	0.8000	0.4000	0.7500	0.8307	0.4006
0.2500	0.4000	0.4500	0.2500	0.3820	0.4446
0.7500	0.4000	0.4500	0.7500	0.3819	0.4446
0.0000	0.0000	0.5000	1.0000	0.0277	0.5096
0.5000	0.0000	0.5000	0.5000	0.0276	0.5096
0.0000	0.6000	0.9500	1.0000	0.6176	0.9442
0.5000	0.6000	0.9500	0.5000	0.6175	0.9442
0.0000	0.2000	0.9000	0.0000	0.1700	0.9004
0.5000	0.2000	0.9000	0.5000	0.1699	0.9003
0.0000	0.8000	0.8500	0.0000	0.8317	0.8475
0.5000	0.8000	0.8500	0.5000	0.8318	0.8475
0.0000	0.4000	0.8000	0.0000	0.3895	0.7948
0.5000	0.4000	0.8000	0.5000	0.3894	0.7948
0.0000	0.0000	0.7500	0.0000	0.9895	0.7519
0.5000	0.0000	0.7500	0.5000	0.9896	0.7519
0.0000	0.6000	0.7000	0.0000	0.6261	0.7051
0.5000	0.6000	0.7000	0.5000	0.6260	0.7050
0.0000	0.2000	0.6500	0.0001	0.1770	0.6530

0.5000	0.2000	0.6500	0.5000	0.1769	0.6530
0.0000	0.8000	0.6000	1.0000	0.8315	0.6037
0.5000	0.8000	0.6000	0.5000	0.8315	0.6037
0.0000	0.4000	0.5500	1.0000	0.3782	0.5415
0.5000	0.4000	0.5500	0.5000	0.3781	0.5415
0.5000	0.3000	0.0250	0.7500	0.3002	0.9724
0.0000	0.3000	0.0250	0.2500	0.3003	0.9724
0.5000	0.9000	0.0750	0.7501	0.8778	0.9273
0.0000	0.9000	0.0750	0.2501	0.8777	0.9273
0.0000	0.5000	0.1250	0.7499	0.4949	0.8748
0.5000	0.5000	0.1250	0.2500	0.4949	0.8748
0.0000	0.1000	0.1750	0.7501	0.1083	0.8270
0.5000	0.1000	0.1750	0.2500	0.1083	0.8270
0.5000	0.7000	0.2250	0.2500	0.6915	0.7774
0.0000	0.7000	0.2250	0.7500	0.6916	0.7775
0.5000	0.3000	0.2750	0.2500	0.3025	0.7213
0.0000	0.3000	0.2750	0.7500	0.3025	0.7213
0.0000	0.9000	0.3250	0.7499	0.8927	0.6746
0.5000	0.9000	0.3250	0.2500	0.8926	0.6746
0.0000	0.5000	0.3750	0.7500	0.5020	0.6242
0.5000	0.5000	0.3750	0.2499	0.5020	0.6242
0.0000	0.1000	0.4250	0.7500	0.1264	0.5705
0.5000	0.1000	0.4250	0.2500	0.1264	0.5705
0.0000	0.7000	0.4750	0.2500	0.6999	0.5326
0.5000	0.7000	0.4750	0.7500	0.6999	0.5326
0.7500	0.3000	0.9750	0.5000	0.2994	0.0315
0.2500	0.3000	0.9750	0.0000	0.2995	0.0315
0.7500	0.9000	0.9250	0.5000	0.8752	0.0702
0.2500	0.9000	0.9250	1.0000	0.8752	0.0702
0.7500	0.5000	0.8750	0.0000	0.4976	0.1234
0.2500	0.5000	0.8750	0.5000	0.4976	0.1234
0.7500	0.1000	0.8250	0.0001	0.1060	0.1743
0.2500	0.1000	0.8250	0.5001	0.1060	0.1743
0.2500	0.7000	0.7750	0.5000	0.6969	0.2212
0.7500	0.7000	0.7750	0.0000	0.6969	0.2212
0.2500	0.3000	0.7250	0.4999	0.3085	0.2771
0.7500	0.3000	0.7250	1.0000	0.3085	0.2771
0.7500	0.9000	0.6750	1.0000	0.8915	0.3268
0.2500	0.9000	0.6750	0.5000	0.8915	0.3268
0.7500	0.5000	0.6250	0.0000	0.5060	0.3753
0.2500	0.5000	0.6250	0.5000	0.5060	0.3753
0.7500	0.1000	0.5750	0.0000	0.1212	0.4274
0.2500	0.1000	0.5750	0.5000	0.1212	0.4274
0.2500	0.7000	0.5250	0.0001	0.6997	0.4729
0.7500	0.7000	0.5250	0.5001	0.6996	0.4729
0.2500	0.3000	0.0250	0.2500	0.2417	0.0310

0.7500	0.3000	0.0250	0.7500	0.2418	0.0310
0.2500	0.9000	0.0750	0.2500	0.8990	0.0820
0.7500	0.9000	0.0750	0.7500	0.8990	0.0820
0.2500	0.5000	0.1250	0.2499	0.4715	0.1097
0.7500	0.5000	0.1250	0.7499	0.4717	0.1096
0.2500	0.1000	0.1750	0.2500	0.0817	0.1823
0.7500	0.1000	0.1750	0.7500	0.0815	0.1823
0.2500	0.7000	0.2250	0.2500	0.6511	0.2158
0.7500	0.7000	0.2250	0.7500	0.6511	0.2157
0.2500	0.3000	0.2750	0.2500	0.3264	0.2880
0.7500	0.3000	0.2750	0.7500	0.3264	0.2880
0.2500	0.9000	0.3250	0.2500	0.8983	0.3162
0.7500	0.9000	0.3250	0.7499	0.8981	0.3161
0.2500	0.5000	0.3750	0.2500	0.5492	0.3832
0.7500	0.5000	0.3750	0.7500	0.5493	0.3832
0.2500	0.1000	0.4250	0.2500	0.1123	0.4161
0.7500	0.1000	0.4250	0.7500	0.1123	0.4161
0.2500	0.7000	0.4750	0.2500	0.7657	0.4733
0.7500	0.7000	0.4750	0.7500	0.7660	0.4733
0.5000	0.5000	0.0000	0.4998	0.5016	0.0097
0.0000	0.5000	0.0000	0.9999	0.5017	0.0097
0.0000	0.1000	0.0500	0.0000	0.0740	0.0429
0.5000	0.1000	0.0500	0.5000	0.0741	0.0429
0.5000	0.7000	0.1000	0.4999	0.6565	0.0835
0.0000	0.7000	0.1000	1.0000	0.6565	0.0835
0.5000	0.3000	0.1500	0.5001	0.3251	0.1627
0.0000	0.3000	0.1500	0.0000	0.3251	0.1626
0.0000	0.9000	0.2000	0.0000	0.9145	0.2078
0.5000	0.9000	0.2000	0.4999	0.9145	0.2078
0.0000	0.5000	0.2500	0.0000	0.4781	0.2389
0.5000	0.5000	0.2500	0.5000	0.4781	0.2389
0.0000	0.1000	0.3000	0.9999	0.1003	0.3025
0.5000	0.1000	0.3000	0.4999	0.1003	0.3025
0.5000	0.7000	0.3500	0.5000	0.7253	0.3661
0.0000	0.7000	0.3500	0.0000	0.7253	0.3661
0.5000	0.3000	0.4000	0.5000	0.2826	0.3879
0.0000	0.3000	0.4000	0.9999	0.2826	0.3879
0.5000	0.9000	0.4500	0.5000	0.8880	0.4461
0.0000	0.9000	0.4500	0.0000	0.8881	0.4461
0.5000	0.8000	0.0250	0.5000	0.8021	0.0177
0.0000	0.8000	0.0250	0.0000	0.8022	0.0177
0.0000	0.4000	0.0750	0.9999	0.3163	0.0880
0.5000	0.4000	0.0750	0.4999	0.3162	0.0880
0.0000	0.0000	0.1250	1.0000	0.9959	0.1245
0.5000	0.0000	0.1250	0.5000	0.9959	0.1245
0.5000	0.6000	0.1750	0.5000	0.6656	0.1632

0.0000	0.6000	0.1750	0.0001	0.6656	0.1632
0.5000	0.2000	0.2250	0.5000	0.1847	0.2295
0.0000	0.2000	0.2250	0.9999	0.1847	0.2295
0.5000	0.8000	0.2750	0.4999	0.7659	0.2775
0.0000	0.8000	0.2750	0.0000	0.7659	0.2775
0.5000	0.4000	0.3250	0.5000	0.4707	0.3185
0.0000	0.4000	0.3250	1.0000	0.4707	0.3185
0.0000	0.0000	0.3750	0.9997	0.0042	0.3759
0.5000	0.0000	0.3750	0.5002	0.0042	0.3758
0.0000	0.6000	0.4250	1.0000	0.5419	0.4346
0.5000	0.6000	0.4250	0.5001	0.5419	0.4346
0.5000	0.2000	0.4750	0.5000	0.2613	0.4707
0.0000	0.2000	0.4750	0.0000	0.2613	0.4707
0.0000	0.3000	0.9750	0.0001	0.2342	0.9722
0.5000	0.3000	0.9750	0.5001	0.2342	0.9722
0.0000	0.9000	0.9250	0.0000	0.8901	0.9163
0.5000	0.9000	0.9250	0.5000	0.8901	0.9163
0.0000	0.5000	0.8750	1.0000	0.4519	0.8825
0.5000	0.5000	0.8750	0.5000	0.4513	0.8825
0.0000	0.1000	0.8250	0.0001	0.1060	0.8159
0.5000	0.1000	0.8250	0.5001	0.1059	0.8159
0.0000	0.7000	0.7750	0.0000	0.6756	0.7883
0.5000	0.7000	0.7750	0.5001	0.6755	0.7883
0.0000	0.3000	0.7250	0.0000	0.3493	0.7162
0.5000	0.3000	0.7250	0.5000	0.3494	0.7164
0.0000	0.9000	0.6750	1.0000	0.9168	0.6826
0.5000	0.9000	0.6750	0.5000	0.9167	0.6826
0.0000	0.5000	0.6250	0.9999	0.5290	0.6105
0.5000	0.5000	0.6250	0.4999	0.5290	0.6105
0.0000	0.1000	0.5750	0.0000	0.1039	0.5825
0.5000	0.1000	0.5750	0.5000	0.1039	0.5825
0.0000	0.7000	0.5250	0.0000	0.7580	0.5315
0.5000	0.7000	0.5250	0.5000	0.7580	0.5315
0.2500	0.5000	0.5000	0.2500	0.4981	0.5105
0.7500	0.5000	0.5000	0.7499	0.4981	0.5105
0.2500	0.1000	0.9500	0.2501	0.1111	0.9459
0.7500	0.1000	0.9500	0.7501	0.1111	0.9459
0.2500	0.7000	0.9000	0.2500	0.7184	0.8872
0.7500	0.7000	0.9000	0.7500	0.7184	0.8872
0.2500	0.3000	0.8500	0.2499	0.2750	0.8661
0.7500	0.3000	0.8500	0.7501	0.2750	0.8661
0.2500	0.9000	0.8000	0.2500	0.8996	0.8027
0.7500	0.9000	0.8000	0.7500	0.8995	0.8027
0.7500	0.5000	0.7500	0.7501	0.5211	0.7393
0.2500	0.5000	0.7500	0.2500	0.5211	0.7393
0.2500	0.1000	0.7000	0.2500	0.0846	0.7077

0.7500	0.1000	0.7000	0.7500	0.0846	0.7077
0.2500	0.7000	0.6500	0.2500	0.6722	0.6639
0.7500	0.7000	0.6500	0.7500	0.6722	0.6639
0.2500	0.3000	0.6000	0.2500	0.3453	0.5835
0.7500	0.3000	0.6000	0.7500	0.3454	0.5835
0.7500	0.9000	0.5500	0.7501	0.9258	0.5441
0.2500	0.9000	0.5500	0.2500	0.9258	0.5440
0.7500	0.8000	0.9750	0.7500	0.7372	0.9705
0.2500	0.8000	0.9750	0.2500	0.7372	0.9705
0.7500	0.4000	0.9250	0.7500	0.4576	0.9340
0.2500	0.4000	0.9250	0.2499	0.4576	0.9340
0.7500	0.0000	0.8750	0.7500	0.9959	0.8760
0.2500	0.0000	0.8750	0.2500	0.9959	0.8760
0.2500	0.6000	0.8250	0.2500	0.5257	0.8178
0.7500	0.6000	0.8250	0.7501	0.5257	0.8178
0.2500	0.2000	0.7750	0.2500	0.2308	0.7771
0.7500	0.2000	0.7750	0.7501	0.2308	0.7771
0.2500	0.8000	0.7250	0.2500	0.8156	0.7299
0.7500	0.8000	0.7250	0.7500	0.8156	0.7299
0.7500	0.4000	0.6750	0.7499	0.3319	0.6633
0.2500	0.4000	0.6750	0.2502	0.3320	0.6633
0.7500	0.0000	0.6250	0.7500	0.0058	0.6251
0.2500	0.0000	0.6250	0.2500	0.0059	0.6251
0.7500	0.6000	0.5750	0.7499	0.6852	0.5892
0.2500	0.6000	0.5750	0.2499	0.6853	0.5892
0.7500	0.2000	0.5250	0.7500	0.1975	0.5178
0.2500	0.2000	0.5250	0.2500	0.1975	0.5178
